# SYNCRIP drives ferroptosis resistance and metabolic activation via SIRT1 and HK2 in glioblastoma

**DOI:** 10.7150/ijbs.127096

**Published:** 2026-07-13

**Authors:** Hyeon Ji Kim, Hyo-Jin Song, Yu Gyung Kim, Mingyu Kang, Tae-Jun Kim, Jin-Seok Byun, Do-Yeon Kim

**Affiliations:** 1 Department of Pharmacology, School of Dentistry, Kyungpook National University, Daegu 41940, Republic of Korea.; 2 Division of Cancer Biology, Research Institute, National Cancer Center, Goyang 10408, Gyeonggi-do, Republic of Korea.; 3 Division of Radiation Biomedical Research, Korea Institute of Radiological and Medical Sciences, Seoul 01812, Republic of Korea.; 4 Department of Oral Medicine, School of Dentistry, Kyungpook National University, Daegu 41940, Republic of Korea.

**Keywords:** SYNCRIP, ferroptosis, SIRT1, IRES, HK2, transcription

## Abstract

Synaptotagmin-binding cytoplasmic RNA-interacting protein (SYNCRIP) is an RNA-binding protein (RBP) implicated in the pathogenesis of various cancers through involvement in regulating multiple cellular processes. Notably, this study identified that SYNCRIP expression is significantly elevated in glioblastoma (GBM) and is associated with poor prognosis and tumor progression. Mechanistically, SYNCRIP upregulates SIRT1 expression at both the transcriptional and post-transcriptional levels by stabilizing *SIRT1* mRNA. Meanwhile, loss of SYNCRIP leads to reduced SIRT1 expression, accumulation of reactive oxygen species (ROS), and induction of ferroptosis. Notably, restoration of SIRT1 rescues cells from ferroptotic cell death, supporting the critical role of SIRT1 in SYNCRIP-mediated ferroptosis resistance. SYNCRIP also enhances hexokinase 2 (HK2) expression through transcriptional activation and internal ribosome entry site (IRES)-mediated translation, thereby promoting glycolytic activity in GBM. Furthermore, depletion of SYNCRIP results in mitochondrial dysfunction and impairs GBM cell migration and invasion by downregulating epithelial-mesenchymal transition (EMT)-associated factors. Collectively, these findings suggest that SYNCRIP is a key regulator of GBM progression by maintaining metabolic homeostasis and ferroptosis resistance, highlighting SYNCRIP as a potential therapeutic target in GBM.

## Introduction

Glioblastoma (GBM) is the most aggressive and lethal primary brain tumor, with a median survival of less than 15 months despite advances in treatment strategies such as surgical resection, radiation therapy, and chemotherapy 1, 2. The poor prognosis of GBM is largely attributable to the highly infiltrative nature, extensive heterogeneity, and strong resistance to conventional therapies associated with the disorder. Therefore, identifying novel molecular targets and therapeutic strategies is essential for improving patient outcomes.

Ferroptosis, a recently characterized form of regulated cell death, has emerged as a potential therapeutic target in GBM. Unlike apoptosis or necrosis, ferroptosis is driven by the accumulation of lipid peroxides resulting from impaired antioxidant defenses 3. This process is mainly regulated by glutathione peroxidase 4 (GPX4), which neutralizes lipid peroxides, and the Xc- system of cystine/glutamate antiporters, which maintains intracellular glutathione (GSH) levels 4. Several studies have demonstrated that inducing ferroptosis through genetic or pharmacological inhibition of these pathways effectively suppresses tumor growth, making ferroptosis a promising avenue for GBM therapy 5-7.

Sirtuin 1 (SIRT1), a member of the NAD^+^-dependent deacetylase family, is a critical regulator of cellular homeostasis that controls oxidative stress, lipid metabolism, and mitochondrial function 8. Recent studies suggest that SIRT1 plays a key role in regulating ferroptosis through multiple mechanisms, including the activation of nuclear factor erythroid 2-related factor 2 (NRF2), a master regulator of antioxidant defense 9. NRF2 enhances the expression of genes involved in glutathione biosynthesis and the detoxification of lipid peroxidation products 10. Additionally, SIRT1 has been reported to deacetylate and inhibit p53, a tumor suppressor that promotes ferroptosis under certain conditions 11. Despite these insights, the precise regulatory mechanisms governing SIRT1 expression in GBM remain largely unexplored.

RNA-binding proteins (RBPs) are critical regulators of post-transcriptional gene expression, influencing pre-mRNA splicing, mRNA stability, and translation 12. Synaptotagmin-binding cytoplasmic RNA-interacting protein (SYNCRIP), also known as heterogeneous nuclear ribonucleoprotein Q (hnRNP Q), is an RBP implicated in diverse cellular processes 13. Recent studies have highlighted the role of RBPs in ferroptosis regulation through post-transcriptional control of *SLC7A11* mRNA. For instance, NKAP protects GBM cells from ferroptosis by promoting m6A-dependent splicing of *SLC7A11* mRNA, whereas DAZAP1 promotes ferroptosis resistance by binding to *SLC7A11* mRNA and regulating the subsequent stability and expression 14, 15. Although SYNCRIP has been implicated in tumorigenesis across several cancer types, the role of SYNCRIP in GBM pathogenesis and ferroptosis regulation remains largely unexplored.

In addition to conventional cap-dependent translation, a subset of mRNAs can be translated via cap-independent mechanisms mediated by internal ribosome entry sites (IRESs). IRES-mediated translation enables selective protein synthesis when cap-dependent translation is suppressed under specific stress conditions, including oxidative stress, thereby supporting cellular survival and metabolic adaptation 16, 17.

Thus, this study demonstrated that SYNCRIP depletion in GBM reduces both *SIRT1* transcription and mRNA stability, thereby decreasing NRF2 expression and impairing antioxidant capacity. Additionally, SYNCRIP knockdown suppresses IRES-mediated translation of the glycolytic enzyme hexokinase 2 (HK2), thereby promoting ferroptosis under stress conditions. These findings reveal a previously unrecognized mechanism through which SYNCRIP confers resistance to ferroptosis in GBM and suggest that targeting SYNCRIP may represent a promising therapeutic strategy for this devastating disease.

## Results

### SYNCRIP promotes proliferation and tumorigenesis in GBM

**Figure 1:**
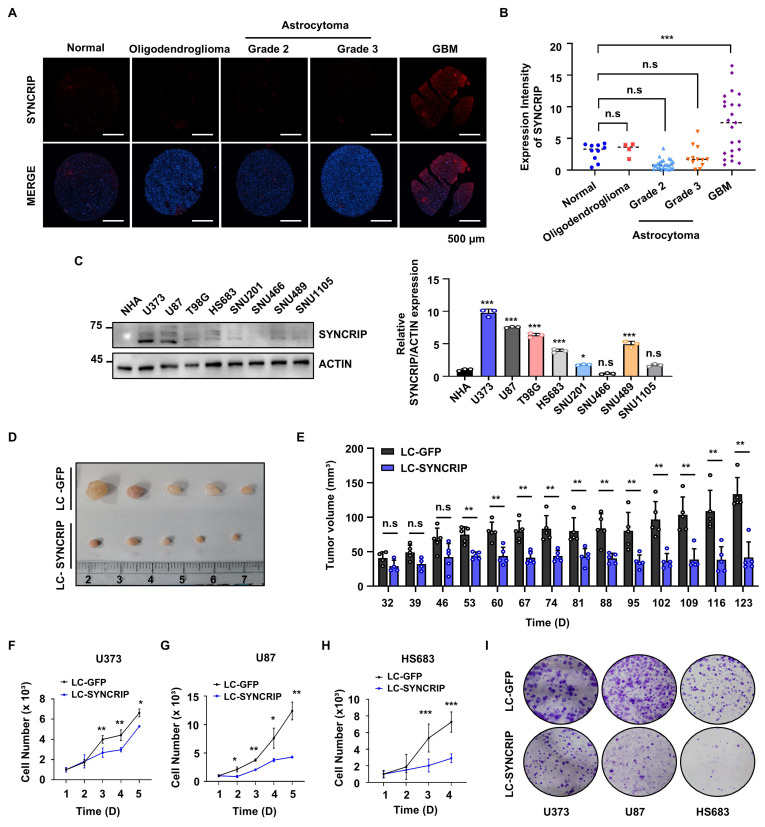
** Loss of SYNCRIP impairs tumorigenesis in GBM. A**. Immunofluorescence staining of a tissue microarray containing normal brain tissues and glioma tissues at various stages of progression (SYNCRIP, red; DAPI, blue). Scale bar, 500 µm. **B**. Quantification of SYNCRIP staining intensity in brain tissue microarrays (normal, n = 10; oligodendroglioma, n = 4; astrocytoma grade 2, n = 26; astrocytoma grade 3, n = 12; GBM, n = 24). Data are presented as the mean ± standard error of the mean (SEM). **C**. Western blot analysis of SYNCRIP expression in normal human astrocytes (NHAs) and GBM cell lines (top). Densitometric quantification of SYNCRIP expression was analyzed (bottom; n = 3). **D.** Representative images of nude mice and xenografts on day 124 post-injection (n = 5). **E**. Tumor growth curves showing average tumor volumes over 123 days. An unpaired two-tailed Student's t-test was used to assess statistical significance. Data are presented as the mean ± SEM. **F-H**. Cell counting assays in control and SYNCRIP-deficient U373 cells (**F**; n = 4), U87 cells (**G**; n = 4), and HS683 cells (**H**; n = 4). Data are presented as mean ± standard deviation (SD). **I**. Colony formation assay comparing control and SYNCRIP-deficient GBM cells. For all statistical tests: *, *P* < 0.05; **, *P* < 0.01; ***, *P* < 0.001; n.s., not significant.

Previous studies have reported that increased SYNCRIP expression is associated with poor prognosis in several cancers 18, 19. Thus, using the Gene Expression Profiling Interactive Analysis (GEPIA) database 20, we observed that SYNCRIP expression was significantly elevated in multiple tumor types, including GBM, compared with normal tissues (Figure S1A) 21. Furthermore, we found that high SYNCRIP expression was significantly associated with poorer overall survival in patients with GBM, as demonstrated by Kaplan-Meier analysis (Figure S1B). Notably, histological analyses revealed that SYNCRIP expression was enriched in GBM tissues compared with lower-grade gliomas and normal brain tissues, suggesting a GBM-specific oncogenic role for SYNCRIP in tumor progression (Figure 1A-B). Consistently, SYNCRIP expression levels were significantly elevated in GBM cell lines compared with normal human astrocytes (NHAs) (Figure 1C).

To investigate the function of SYNCRIP in GBM, SYNCRIP-knockout (KO) cells were generated using the CRISPR/Cas9 system. To minimize off-target effects and cell type-specific phenomena, three GBM cell lines (U373, U87, and HS683) with high endogenous SYNCRIP expression were selected for gene editing. To assess the impact of SYNCRIP depletion on tumorigenesis *in vivo*, we performed xenograft experiments using U373 cells stably lacking SYNCRIP. Tumor growth was significantly suppressed in SYNCRIP-deficient xenografts, as evidenced by reduced tumor size and lower growth rates compared with control groups (Figures 1D-E and S1C). SYNCRIP deficiency *in vitro* also led to a marked reduction in cell proliferation and colony formation across GBM cell lines (Figure 1F-I). Consistent with these findings, BrdU incorporation assays further confirmed that loss of SYNCRIP markedly reduced proliferation rates (Figure S1D). Collectively, these results suggest that SYNCRIP plays a critical role in promoting tumor growth and proliferation in GBM.

### SYNCRIP deficiency promotes reactive oxygen species (ROS) accumulation and ferroptosis

**Figure 2:**
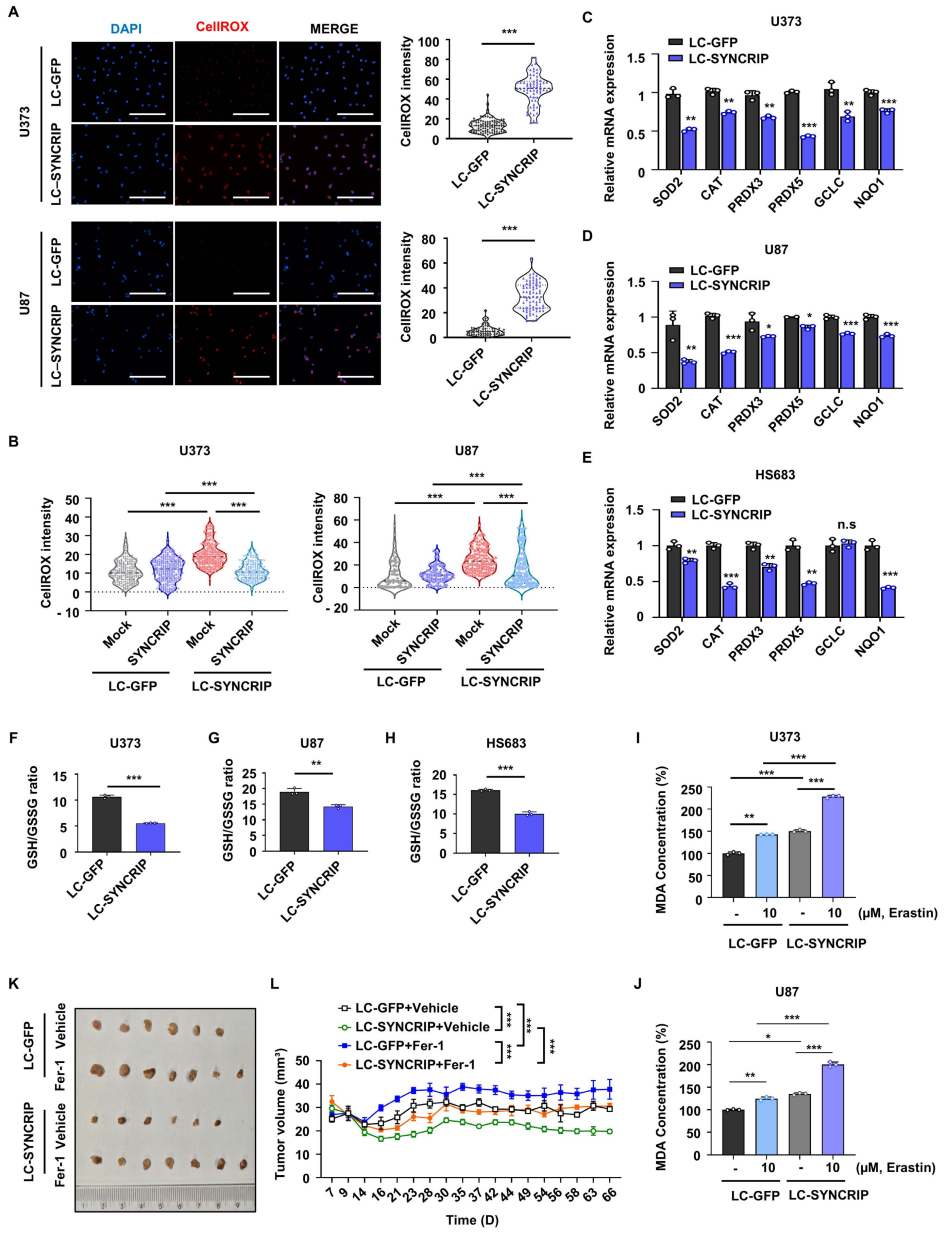
** SYNCRIP deficiency induces lipid peroxidation. A**. Fluorescence imaging of reactive oxygen species (ROS) accumulation in SYNCRIP-deficient U373 (n = 80) and U87 (n = 60) cells using CellROX (red) with DAPI nuclear counterstaining (blue) (left). Quantification of CellROX fluorescence intensity relative to the control (right). Scale bar, 200 µm. Student's t-test was used to assess statistical significance. Data are presented as the mean ± SD. **B**. Control (LC-GFP) and SYNCRIP-deficient U373 (n = 200-300 per group) and U87 (n = 100-200 per group) cells were transfected with EGFP-mock (Mock) or EGFP-SYNCRIP (SYNCRIP), and ROS levels were assessed by CellROX staining. One-way analysis of variance (ANOVA) with Dunnett's post hoc test was used for statistical analysis. **C-E**. Relative mRNA expressions of the indicated genes in control and SYNCRIP-deficient U373 (**C**), U87 (**D**), and HS683 (**E**) cells. **F-H**. GSH/GSSG ratios in control and SYNCRIP KO U373 (**F**), U87 (**G**), and HS683 (**H**) cells. Student's t-test was used to assess statistical significance (n = 3; *, *P* < 0.05; **, *P* < 0.01; ***, *P* < 0.001; n.s., nonsignificant). Data are presented as the mean ± SEM. **I-J**. Measurement of MDA concentrations in U373 (**I**) and U87 (**J**) cells. Erastin (10 µM) was used as a positive control. One-way ANOVA with Dunnett's post hoc test was performed (n = 3; *, *P* < 0.05; **, *P* < 0.01; ***, *P* < 0.001). **K**. Representative images of tumors from xenograft models derived from LC-GFP and LC-SYNCRIP cells, with or without ferrostatin-1 (Fer-1) treatment. Tumors were collected 66 days after injection (DMSO, n = 6; Fer-1, n = 7). **L**. Tumor growth curves from the xenografts show that Fer-1 treatment rescues tumor growth in SYNCRIP-deficient tumors (DMSO, n = 6; Fer-1, n = 7). Data are presented as the mean ± SEM. Statistical significance was assessed using two-way ANOVA with Šidák's multiple comparisons test (***, *P* < 0.001).

Oxidative stress, caused by elevated levels of ROS, can inhibit tumor growth by inducing cell death or suppressing proliferation 22. Therefore, to investigate whether SYNCRIP modulates the oxidative stress response in GBM, we measured intracellular ROS levels using CellROX staining. SYNCRIP-deficient GBM cells exhibited a pronounced increase in ROS accumulation compared with controls (Figures 2A and S2A). Reintroduction of SYNCRIP into KO cells reversed this phenotype, indicating that SYNCRIP is critical for maintaining redox homeostasis in GBM (Figures 2B and S2B). Consistently, SYNCRIP depletion reduced the expression of several antioxidant-related genes, including SOD2, CAT, PRDX3, PRDX5, and NQO1 (Figures 2C-E and S2C-E).

Since glutamate-cysteine ligase catalytic subunit (GCLC) and NAD(P)H quinone dehydrogenase 1 (NQO1) are key regulators of GSH biosynthesis and oxidative stress resistance, we hypothesized that SYNCRIP might influence ferroptosis through modulation of glutathione metabolism 23, 24. Notably, SYNCRIP KO cells showed a significantly reduced GSH/GSSG ratio, indicating compromised antioxidant capacity (Figure 2F-H). Hence, to assess whether SYNCRIP depletion enhances susceptibility to ferroptosis directly, we measured malondialdehyde (MDA) levels, a key marker of lipid peroxidation. MDA levels were markedly increased in SYNCRIP-deficient cells, providing strong evidence that loss of SYNCRIP sensitizes GBM cells to ferroptotic cell death. Furthermore, treatment with the ferroptosis inducer erastin (10 µM) led to an additional increase in MDA levels in SYNCRIP KO cells compared with controls, whereas treatment with the ferroptosis inhibitor ferrostatin-1 (Fer-1; 50 µM) reduced MDA levels in SYNCRIP-deficient cells (Figures 2I-J and S2F-G). These results suggest that lipid peroxidation induced by SYNCRIP depletion is associated with ferroptosis. Consistent with these findings, *in vivo* treatment with ferrostatin-1 (Fer-1) partially restored tumor growth inhibited by SYNCRIP deficiency in xenograft models (Figure 2K-L). This result indicates that ferroptosis contributes to the antitumor effect induced by SYNCRIP depletion.

Interestingly, HS683 cells, which naturally express low levels of SYNCRIP, were more sensitive to erastin-induced ferroptosis (Figure S3A-C). SYNCRIP-deficient cells exhibited dose-dependent cell death upon erastin treatment (Figure S3D-F). Meanwhile, immunoblotting revealed that TFRC expression increased following erastin treatment (Figure S4A-B), whereas Fer-1 treatment reduced TFRC levels (Figure S4C-D), further supporting a role for SYNCRIP in regulating ferroptosis.

Additionally, treatment with Fer-1 partially reduced intracellular ROS accumulation, as determined by CellROX staining (Figure S5A-C). This finding suggests that suppressing ferroptosis-related oxidative stress can partially mitigate the redox imbalance caused by SYNCRIP deficiency.

Since elevated ROS can trigger multiple forms of cell death, we further investigated whether SYNCRIP deficiency induces apoptosis. Annexin V/PI staining revealed no significant increase in apoptotic or necrotic cell populations in SYNCRIP-deficient U373, U87, and HS683 cells compared with control cells (Figure S6A). Consistently, cleaved caspase-3 expression was not increased under basal conditions (Figure S6B-D). Together, these results indicate that ROS accumulation induced by SYNCRIP deficiency is predominantly associated with ferroptosis rather than apoptosis. Meanwhile, these findings also demonstrate that SYNCRIP protects GBM cells from ferroptosis by maintaining redox balance and antioxidant defense.

### SYNCRIP alleviates ferroptosis by upregulating SIRT1 in GBM

**Figure 3:**
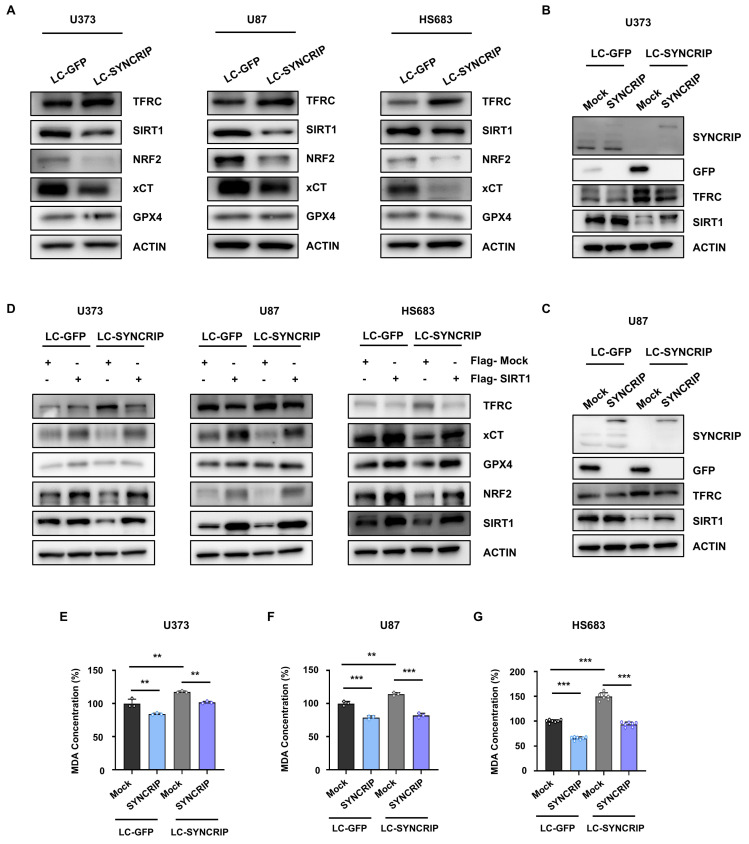
** SYNCRIP mitigates ferroptosis through SIRT1 upregulation in GBM. A.** Immunoblot analysis showing protein expression changes in SYNCRIP-deficient GBM cells. **B-C**. Immunoblot validation of SYNCRIP restoration after transfection with EGFP-mock (Mock) or EGFP-SYNCRIP (SYNCRIP) in control (LC-GFP) and SYNCRIP-deficient (LC-SYNCRIP) U373 (**B**) and U87 (**C**) cells. **D**. Immunoblot analysis of ferroptosis-related proteins after transfection with Flag-Mock or Flag-SIRT1 into control and SYNCRIP-deficient U373, U87, and HS683 cells. Actin was used as a loading control. **E-G**. Measurement of MDA concentrations after SYNCRIP restoration in SYNCRIP-deficient U373 (**E**, n = 3), U87 (**F**, n = 3), and HS683 (**G**, n = 7) cells. Statistical significance was assessed by one-way ANOVA with Dunnett's post hoc test (**, *P* < 0.01; ***, *P* < 0.001).

SIRT1 has previously been identified as a key regulator of ferroptosis 9, 25. Interestingly, analysis of the GEPIA database revealed a positive correlation between SYNCRIP and SIRT1 expression (Figure S4E). To investigate this relationship further, we examined the expression of SIRT1 and downstream ferroptosis-related genes. SIRT1 is known to activate NRF2, a key transcription factor that promotes the expression of antioxidant genes such as *NQO1* 26. Our data showed that SYNCRIP deficiency significantly reduced the expression levels of SIRT1, NRF2, and xCT, while increasing TFRC expression (Figure 3A). Furthermore, SYNCRIP depletion resulted in a marked decrease in intracellular NAD^+^ levels, a crucial cofactor required for the enzymatic activity of the NAD^+^-dependent deacetylase SIRT1 (Figure S4F-H) 27. This reduction in NAD^+^ was accompanied by decreased *SIRT1* mRNA levels, suggesting that SYNCRIP may regulate both the abundance and functional capacity of SIRT1 (Figure S4I).

To further validate the regulatory relationship between SYNCRIP and SIRT1, we performed a rescue experiment by reintroducing SYNCRIP into SYNCRIP KO cells. Notably, restoration of SYNCRIP expression successfully reversed the reduction in SIRT1 levels observed in SYNCRIP-deficient cells, providing strong evidence that SYNCRIP positively regulates SIRT1 expression (Figure 3B-C). Additionally, the increased MDA levels observed upon SYNCRIP depletion were significantly reduced following SYNCRIP restoration (Figure 3E-G), further linking SYNCRIP function to suppression of ferroptosis.

To determine whether the anti-ferroptotic effect of SYNCRIP is mediated through SIRT1, we performed a SIRT1 rescue experiment in SYNCRIP KO cells. Reintroduction of SIRT1 restored xCT and NRF2 expression and suppressed TFRC levels (Figure 3D). Collectively, these findings strongly support the conclusion that SYNCRIP protects GBM cells from ferroptosis by upregulating SIRT1, thereby maintaining redox balance.

### SYNCRIP binds to the *SIRT1* promoter and regulates its transcription

**Figure 4:**
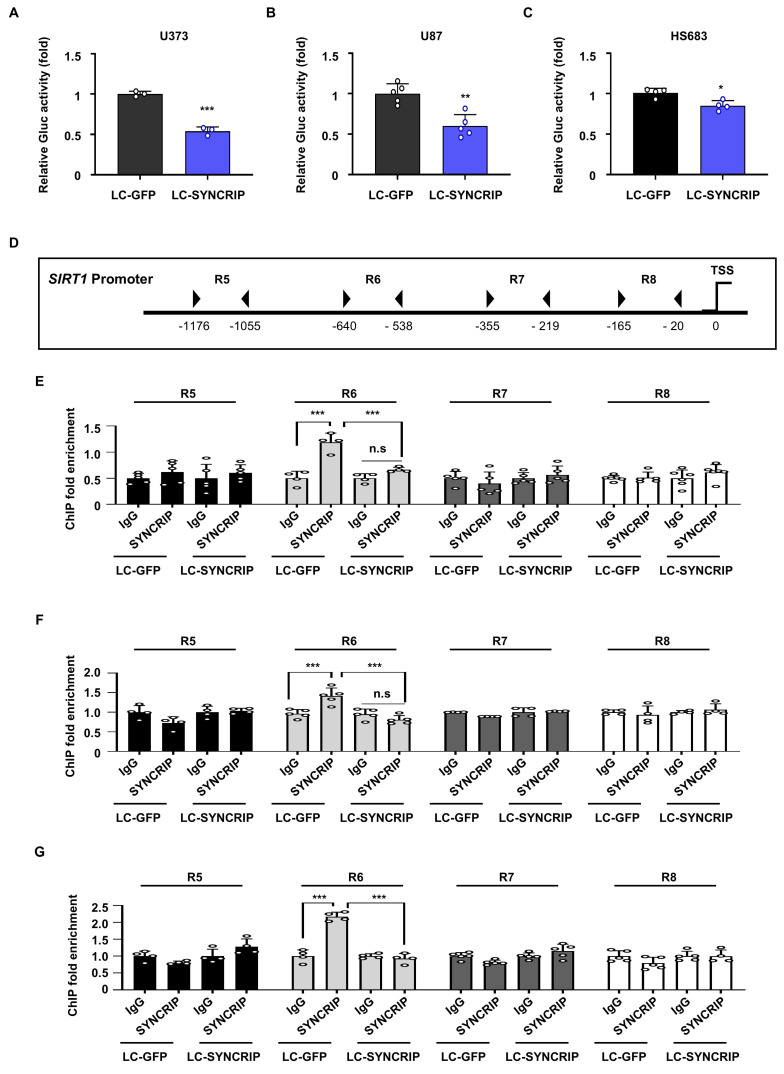
** SYNCRIP interacts with the *SIRT1* promoter to modulate gene transcription. A-C**. Gaussia luciferase (GLUC) reporter assays using *SIRT1* promoter constructs transfected into control and SYNCRIP KO U373 (**A**, n = 3), U87 (**B**, n = 5), and HS683 (**C**, n = 4) cells. Relative luciferase activity is expressed as the GLUC-to-RLUC ratio. Statistical significance was assessed using Student's t-test (*, *P* < 0.05; **, *P* < 0.01; ***, *P* < 0.001). Data are presented as the mean ± SEM. **D**. Schematic diagram of the SIRT1 promoter regions used for ChIP analysis. **E-G**. ChIP fold enrichment of SYNCRIP binding at different regions of the SIRT1 promoter in U373 (**E**), U87 (**F**), and HS683 (**G**) cells. Statistical analysis was performed using two-way ANOVA with Šidák's multiple comparisons test (***, *P* < 0.001). Data are presented as the mean ± SEM (n = 4-6 per group).

After demonstrating that SYNCRIP enhances SIRT1 expression and attenuates ferroptosis, we investigated the molecular mechanisms underlying this regulation. Previous studies have shown that certain RBPs can regulate transcription by binding to promoter regions, as shown for HuR in the regulation of *SIRT1* 28-30. Therefore, to determine whether SYNCRIP exerts transcriptional control over *SIRT1*, we first assessed *SIRT1* promoter activity using a dual-luciferase reporter assay. A significant reduction in *SIRT1* promoter activity was observed in SYNCRIP KO GBM cells compared with controls, suggesting that SYNCRIP positively regulates *SIRT1* transcription (Figure 4A-C).

Subsequently, a chromatin immunoprecipitation (ChIP) assay was conducted to determine whether SYNCRIP binds to the *SIRT1* promoter. Our results demonstrated that SYNCRIP specifically binds to the R6 region (-640 to -538) of the *SIRT1* promoter, indicating a physical interaction between SYNCRIP and the *SIRT1* gene. Notably, SYNCRIP binding was significantly enriched at the R6 region compared with other *SIRT1* promoter regions, reinforcing the specificity of this interaction (Figures 4D-G and S7A-D). To further evaluate the functional significance of the R6 region, additional luciferase assays were performed using promoter fragments lacking this region. Moreover, depletion of SYNCRIP significantly reduced promoter activity in the full-length *SIRT1* promoter construct. However, this reduction was not observed in the upstream and downstream constructs lacking the R6 region, where no significant difference in promoter activity was detected between control and SYNCRIP-deficient cells. These results indicate that SYNCRIP-dependent transcriptional regulation largely depends on the presence of the R6 region (Figure S8A-D). These findings suggest that SYNCRIP acts as a transcriptional regulator of SIRT1 by recognizing and binding to a distinct sequence within the SIRT1 promoter, potentially facilitating transcriptional activation.

### SYNCRIP is involved in the stabilization of *SIRT1* mRNA

**Figure 5:**
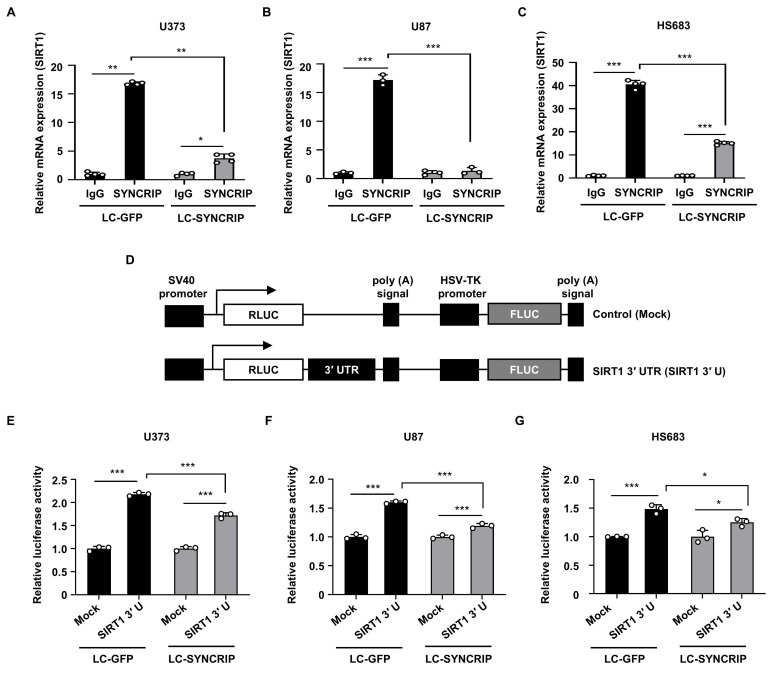
** SYNCRIP increases mRNA stability by binding to the 3' UTR of *SIRT1* mRNA**. **A-C**. RIP assays were conducted using anti-SYNCRIP or non-specific IgG antibodies in U373 (**A**, n = 4), U87 (**B**, n = 3), and HS683 (**C**, n = 4) cells. Statistical analysis was performed using two-way ANOVA with Šidák's multiple comparisons test (*, *P* < 0.05; **, *P* < 0.01; ***, *P* < 0.001). Bars represent the mean ± SEM. **D**. Schematic representation of a reporter plasmid containing the full-length 3' UTR of *SIRT1* mRNA. **E-G**. Relative luciferase activity was expressed as the RLUC/FLUC ratio relative to the control (LC-GFP Mock) in U373 (**E**), U87 (**F**), and HS683 (**G**) cells (n = 3 per group). Data are presented as the mean ± SEM. Two-way ANOVA with Šidák's multiple comparisons test was used for statistical analysis (*, *P* < 0.05; ***, *P* < 0.001).

In addition to a transcriptional role, SYNCRIP has been reported to govern diverse cellular processes as a crucial post-transcriptional regulator. To test whether SYNCRIP regulates the fate of *SIRT1* mRNA, we first performed an RNA immunoprecipitation (RIP) assay. Our data revealed a significant interaction between SYNCRIP protein and *SIRT1* mRNA, which was not observed in SYNCRIP-deficient cells (Figures 5A-C and S9A-B). To further validate the physiological significance of this interaction, we treated GBM cells with actinomycin D (ActD), a transcriptional inhibitor that prevents the synthesis of new mRNA. The results demonstrated that *SIRT1* mRNA exhibited a significantly faster decay rate in the absence of SYNCRIP than in control cells, indicating that SYNCRIP plays a critical role in protecting *SIRT1* mRNA from rapid degradation (Figure S9C-E).

Given the importance of the 3' untranslated region (3' UTR) in mRNA stability 31, we next investigated whether SYNCRIP stabilizes SIRT1 mRNA through interaction with the associated 3' UTR using a luciferase reporter assay containing the *SIRT1* 3' UTR. Luciferase activity was significantly reduced in SYNCRIP-deficient cells, indicating that SYNCRIP enhances *SIRT1* mRNA stability through interaction with the* SIRT1* 3' UTR (Figure 5D-G).

Together, these findings demonstrate that SYNCRIP regulates SIRT1 expression at both the transcriptional and post-transcriptional levels. This dual mechanism highlights the critical role of SYNCRIP in modulating SIRT1 expression and function in GBM.

### SYNCRIP coordinates transcriptional activation and cap-independent translation of HK2

**Figure 6:**
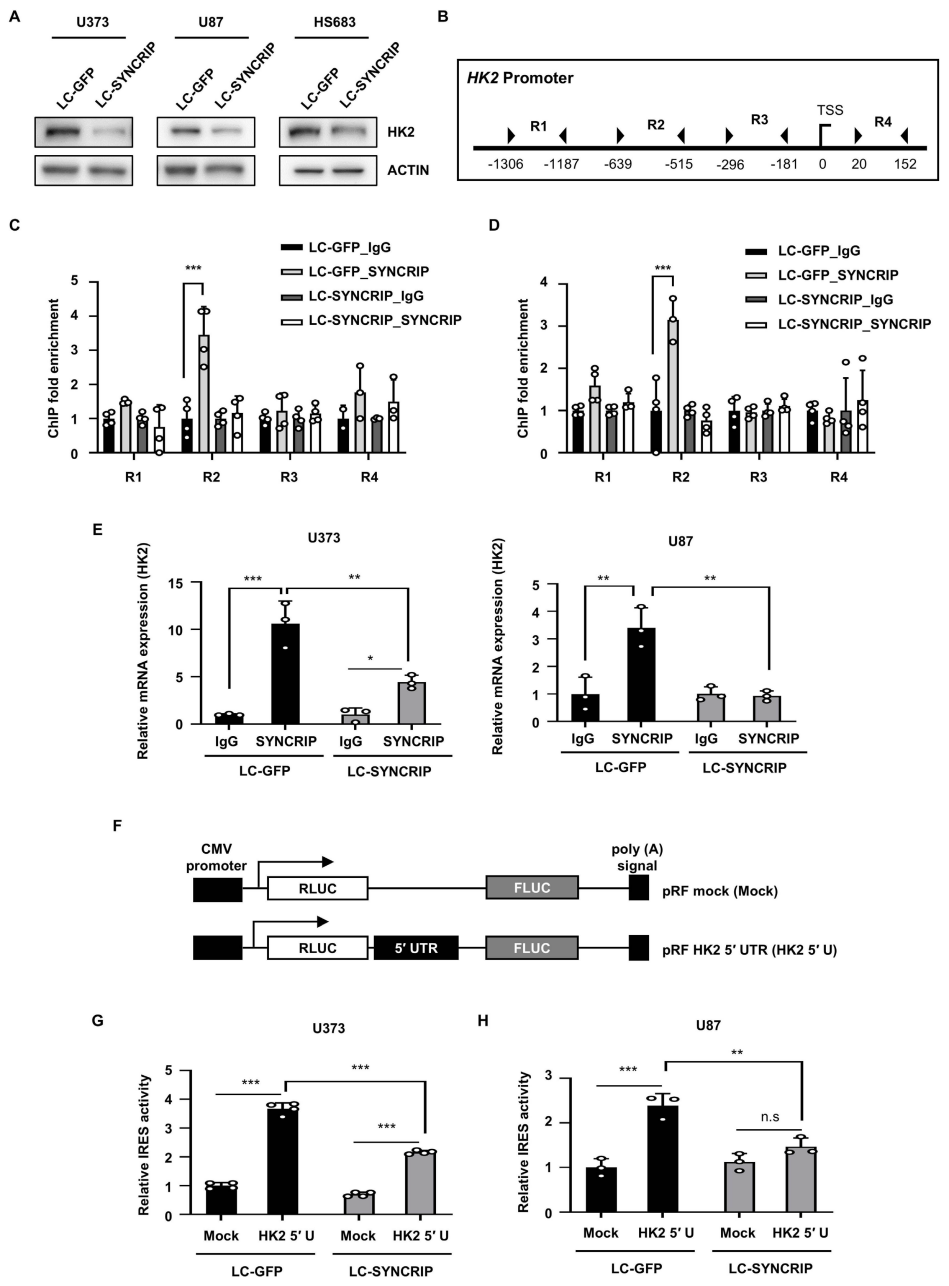
** SYNCRIP regulates HK2 expression via transcriptional activation and IRES-mediated translation. A.** Western blot analysis of HK2 levels in control and SYNCRIP-deficient cells. Actin was used as a loading control.** B**. Schematic representation of the HK2 promoter region used ChIP analysis. **C-D**. ChIP fold enrichment of SYNCRIP binding at different regions of the HK2 promoter in U373 (**C**) and U87 (**D**) cells. Statistical significance was assessed using two-way ANOVA with Šidák's multiple comparisons test (n = 4; ***, *P* < 0.001). **E**. The interaction between SYNCRIP and HK2 mRNA was measured by RNA immunoprecipitation (RIP) assay using non-specific IgG or anti-SYNCRIP antibodies. Two-way ANOVA with Šidák's multiple comparisons test was used for statistical analysis (n = 3; ***, *P* < 0.001). **F**. Schematic representation of the bicistronic reporter plasmid containing the full-length 5′ UTR of HK2 mRNA. **G-H**. Dual luciferase reporter assay results in U373 (**G**, n = 4) and U87 (**H**, n = 3) cells. FLUC/RLUC ratios were normalized to the pRF mock-transfected group. Bars represent the mean ± SEM. Two-way ANOVA with Šidák's multiple comparisons test was used to determine significance (**, *P* < 0.01; ***, *P* < 0.001; n.s, not significant).

SIRT1 has been reported to play a pivotal role in the epithelial-mesenchymal transition (EMT) by regulating glycolysis through modulation of HK2 expression 32, 33. Given that SYNCRIP depletion reduced SIRT1 expression, we examined whether the SYNCRIP-SIRT1 axis contributes to the downstream regulation of HK2 and metabolic reprogramming. To test this hypothesis, we first analyzed HK2 expression in SYNCRIP-deficient cells and found that HK2 levels were significantly lower than in controls (Figures 6A and S10A-B). Interestingly, this reduction in HK2 expression was accompanied by upregulation of GLUT1, an upstream regulator of HK2, suggesting a potential compensatory response to metabolic stress.

To further elucidate the mechanism underlying HK2 regulation by SYNCRIP, we investigated the potential for transcriptional control (Figure S10C-D). ChIP analysis confirmed that SYNCRIP specifically binds to the R2 region (-639 to -515) of the *HK2* promoter (Figures 6B-D and S10E), suggesting that SYNCRIP functions as a transcriptional activator of *HK2* expression.

We also examined the possibility of post-transcriptional regulation of HK2 by SYNCRIP. RIP analysis confirmed that SYNCRIP physically interacts with *HK2* mRNA, and this interaction was not evident in SYNCRIP-deficient cells (Figure 6E). Thus, since HK2 translation is known to be regulated by an IRES mechanism 34, we performed a luciferase assay to assess whether SYNCRIP affects HK2 expression through IRES-mediated translation. The results demonstrated a significant reduction in IRES-driven luciferase activity following SYNCRIP depletion, further supporting the idea that SYNCRIP facilitates cap-independent translation of *HK2* mRNA (Figure 6F-H). Together, these findings suggest that SYNCRIP regulates HK2 expression at both the transcriptional and post-transcriptional levels, thereby likely contributing to the modulation of glycolytic activity in GBM cells.

### SYNCRIP preserves mitochondrial function by suppressing ROS

ROS perform a dual role in cancer, acting as signaling molecules that promote tumor progression at moderate levels but inducing cellular damage when excessively accumulated. Previous studies have shown that uncontrolled accumulation of ROS can severely impair mitochondrial function, leading to metabolic dysfunction and reduced cellular viability 35. Mitochondrial membrane potential (ΔΨm) is a key indicator of mitochondrial integrity, and any reduction in potential is frequently associated with increased ROS levels and compromised mitochondrial activity 36.

**Figure 7:**
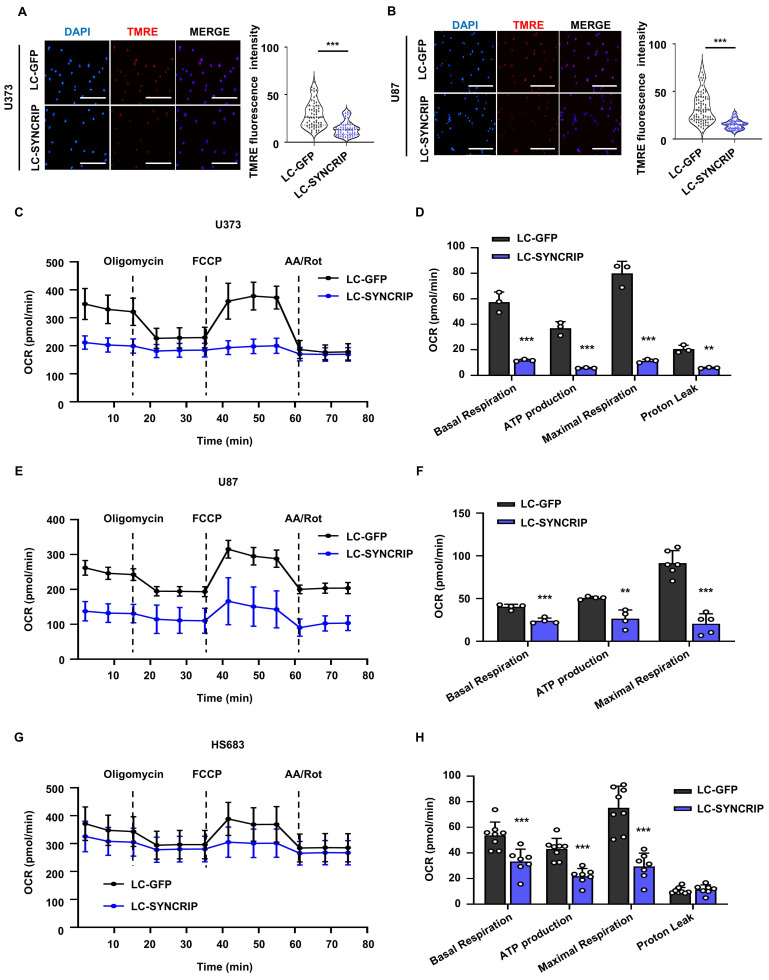
** SYNCRIP silencing impairs mitochondrial function. A-B.** Mitochondrial membrane potential was determined by TMRE staining in control and SYNCRIP-deficient U373 (**A**) and U87 (**B**) cells (n = 10). Data are presented as the mean ± SEM. Student's t-test was used to assess statistical significance (***, *P* < 0.001). **C-H**. OCR was measured over time using a Seahorse XF analyzer in U373 (**C-D**; n = 3), U87 (**E-F**; n = 4), and HS683 (**G-H**; n = 8). Mitochondrial function regulators, including oligomycin, carbonyl cyanide-p-trifluoromethoxyphenylhydrazone (FCCP), and a mixture of rotenone and antimycin A (AA/Rot), were sequentially added at the indicated time points. OCR values were normalized to cell number. Data are shown as the mean ± SEM. Statistical analysis was performed using two-way ANOVA with Šidák's multiple comparisons test (**, *P* < 0.01; ***, *P* < 0.001).

Based on our data finding that SYNCRIP depletion increased ROS levels, we next examined mitochondrial function under SYNCRIP silencing. To assess this, we measured mitochondrial membrane potential (ΔΨm) using tetramethylrhodamine ethyl ester (TMRE) staining. Subsequently, we observed a significant reduction in ΔΨm in SYNCRIP-deficient cells, indicating mitochondrial impairment (Figure 7A-B).

To further elucidate the effect of SYNCRIP depletion on mitochondrial function, mitochondrial bioenergetics were assessed using the Seahorse Mito Stress test. SYNCRIP-deficient cells exhibited a significant reduction in basal respiration, ATP production, and maximal respiratory capacity (Figure 7C-H). These findings are consistent with previous reports linking TMRE loss to decreased oxygen consumption rate (OCR), further supporting the conclusion that SYNCRIP depletion disrupts mitochondrial function and metabolism 37. Collectively, these findings suggest that SYNCRIP plays an essential role in maintaining mitochondrial integrity by preventing excessive accumulation of ROS and preserving cellular bioenergetic capacity.

### SYNCRIP promotes GBM metastasis by enhancing migration and invasion

**Figure 8:**
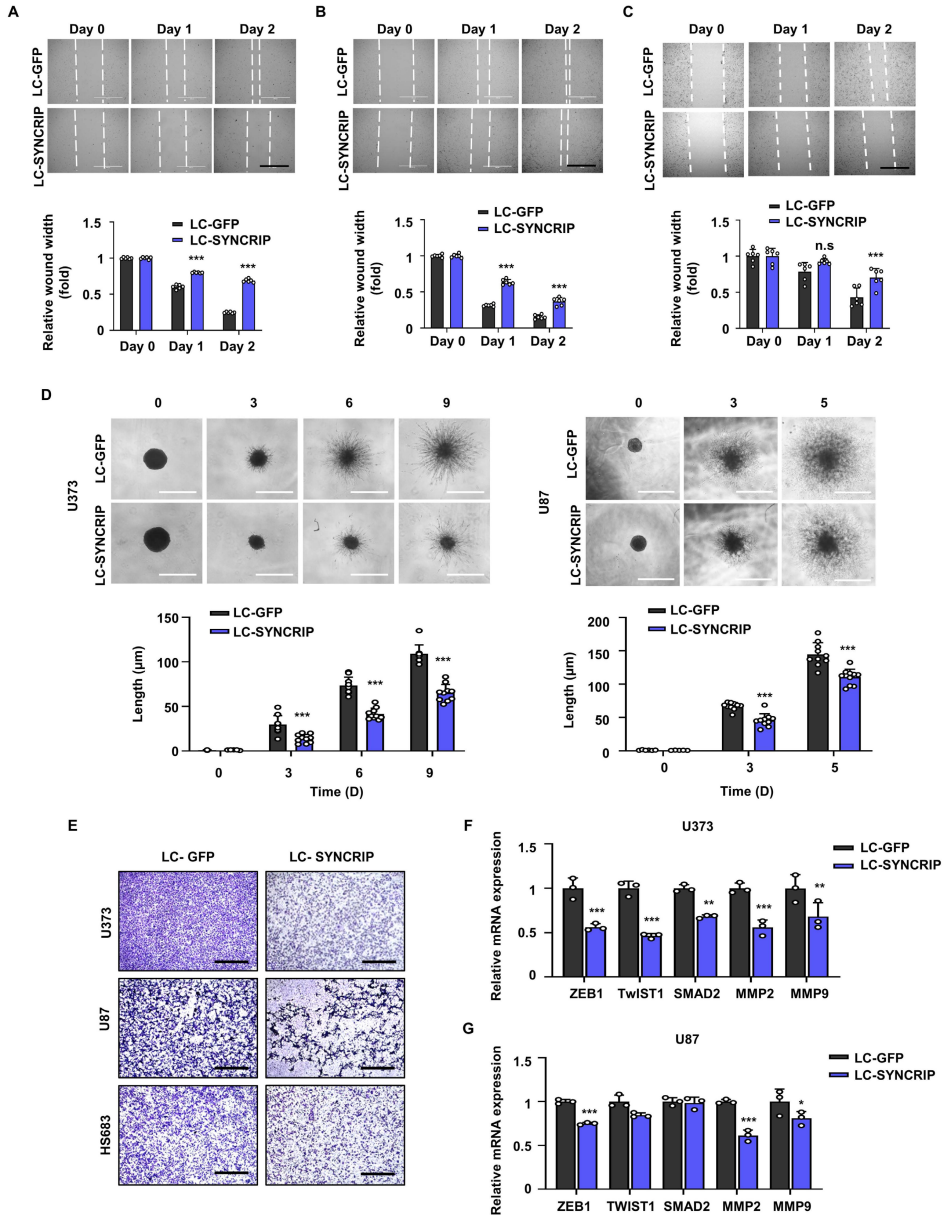
** SYNCRIP promotes the metastasis of glioblastoma. A-C.** Representative images (top) and quantification (bottom) of wound-healing assays in control and SYNCRIP-deficient U373 (**A**), U87 (**B**), and HS683 (**C**) cells. Scale bar, 1000 µm. Data are presented as the mean ± SD. Statistical significance was determined using two-way ANOVA with Šidák's multiple comparisons test (n = 6; ***, *P* < 0.001; n.s., not significant). **D.** Representative images (top) and quantification (bottom) of 3D spheroid invasion assays in control and SYNCRIP-deleted U373 (left) and U87 (right) cells. Scale bars, 500 µm. Statistical significance was determined using two-way ANOVA with Šidák's multiple comparisons test (n = 10; ***, *P* < 0.001; n.s., not significant). **E**. Transwell migration assay showing reduced migratory capacity of in SYNCRIP-deficient cells compared with controls, visualized by crystal violet staining. **F-G**. Relative mRNA expression levels of the indicated genes in U373 (**F**) and U87 (**G**) cells. Data are presented as the mean ± SD. Student's t-test was used to assess statistical significance.

Metastasis is a key feature of GBM that contributes to the associated aggressive nature and poor prognosis of the disorder. Therefore, to investigate whether SYNCRIP influences the metastatic potential of GBM, we performed a wound healing assay to assess cell migration. The results demonstrated that SYNCRIP KO GBM cells exhibited significantly reduced wound-closure rates compared to control cells, suggesting that SYNCRIP facilitates GBM cell migration (Figure 8A-C).

Consistent with these results, SYNCRIP-depleted cells displayed a marked reduction in invasive capacity compared with control cells, as evidenced by the spheroid three-dimensional (3D) invasion assay and Transwell invasion test (Figure 8D-E), further supporting the role of SYNCRIP in promoting GBM invasion.

Since EMT is a crucial process in cancer metastasis, we examined the expression of key EMT-related genes. In SYNCRIP KO U87 cells, we observed a substantial reduction in the expression of ZEB1, MMP2, and MMP9. Similarly, SYNCRIP depletion in U373 cells led to an overall downregulation of EMT-associated markers (Figure 8F-G). Collectively, these results suggest that SYNCRIP enhances the metastatic potential of GBM by promoting cell migration and invasion, likely through regulation of EMT-associated pathways.

## Discussion

SYNCRIP expression is altered in a variety of pathological conditions, including cancer; however, the specific mechanisms underlying SYNCRIP expression in GBM remain unclear 38-40. Subsequently, this study identified SYNCRIP as a key regulator of SIRT1 and the associated downstream glycolytic target enzyme, HK2. This regulatory axis protects GBM cells from lipid peroxidation and oxidative stress, ultimately contributing to tumor progression and metastasis. Given the involvement of RBPs in redox homeostasis, our findings suggest that SYNCRIP may represent a potential therapeutic target.

We demonstrated that SYNCRIP deficiency sensitizes GBM cells to ferroptosis by inducing ROS accumulation, suggesting that ferroptosis is a key mechanism underlying the antitumor phenotype observed in SYNCRIP-deficient cells. However, tumor proliferation is governed by complex, interconnected cellular processes, and our data indicate that oxidative stress alone does not fully account for the observed reduction in proliferative capacity. Since cell proliferation is influenced by multiple regulatory pathways, including metabolic state, mitochondrial function, and redox balance, additional mechanisms beyond ferroptosis likely contribute to this phenotype. Notably, SYNCRIP-deficient cells exhibited mitochondrial dysfunction, including reduced oxygen consumption and decreased mitochondrial membrane potential, indicating broader defects in cellular metabolism. These results suggest that, in addition to ferroptosis, mitochondrial dysfunction and metabolic disturbances may also contribute to the reduced proliferation observed following SYNCRIP depletion.

SIRT1 is known to regulate NRF2, which controls antioxidant- and iron metabolism-related genes, including *xCT* and *GPX4* 41. Consistently, our findings revealed that SYNCRIP depletion reduced the expression of SIRT1, NRF2, and xCT, whereas GPX4 levels remained unchanged (Figure 3A). These results suggest that SYNCRIP regulates ferroptosis indirectly through the SIRT1/NRF2/xCT axis rather than by directly modulating GPX4. Interestingly, restoration of SIRT1 expression both suppressed ferroptosis and increased GPX4 levels, despite the absence of a significant difference in GPX4 expression between SYNCRIP KO and control cells (Figure 3D). These observations underscore the complexity of ferroptosis regulation, suggesting that this process is not solely dependent on GPX4 but instead involves multiple interrelated pathways. Moreover, additional ferroptosis regulators may participate in this process. For example, FSP1 is known to inhibit lipid peroxidation by transforming CoQ10 to an antioxidant form 42. In addition, NRF2 regulates genes involved in iron metabolism such as *ferritin* (*FTL/FTH1*) and *ferroportin* (*SLC40A1*) 43. Thus, SYNCRIP may regulate ferroptosis through broader pathways beyond GPX4, underscoring the need for further investigation to elucidate the precise molecular pathways involved.

To our knowledge, this is the first study to demonstrate that SYNCRIP regulates both the transcription and mRNA stability of SIRT1. Previous reports have noted that *SIRT1* transcription is regulated by p53, Foxo3a, and C/EBPα 44, 45. Our study identified a physical interaction between SYNCRIP and the *SIRT1* promoter, supporting the role of SYNCRIP as a transcriptional regulator (Figure 4). Although the precise binding motif of SYNCRIP within the *SIRT1* 3' UTR remains unidentified, we also confirmed that SYNCRIP binds to the *SIRT1* 3' UTR and affects gene stability (Figure 5). Previous studies have shown that SYNCRIP shuttles between the nucleus and cytoplasm 46, suggesting that the transcriptional and post-transcriptional functions of SYNCRIP are spatially coordinated across distinct cellular compartments. Since Hu-antigen R (HuR) has been shown to stabilize *SIRT1* mRNA by competing with tristetraprolin (TTP) for binding 29, future investigations should determine whether SYNCRIP competes or cooperates with other RBPs, such as HuR or TTP, and whether the interaction between SYNCRIP and the 3' UTR also affects miRNA-mediated regulation of *SIRT1*. Interestingly, the degree of SIRT1 downregulation following SYNCRIP depletion varied across cell lines. In HS683 cells, the reduction was less pronounced than in other GBM cell lines, possibly due to their inherently low baseline expression of SYNCRIP.

SYNCRIP has previously been reported to regulate genes such as *FMR1*, *AURKB*, and *mPer1* via IRES-mediated translation 47-49. Consistent with this, we found that SYNCRIP facilitates IRES-mediated translation of HK2, suggesting a role for SYNCRIP in glycolytic activity. However, whether this occurs independently or in coordination with other translation initiation factors remains unclear and warrants further investigation.

Overall, our findings demonstrate that SYNCRIP employs distinct regulatory mechanisms tailored to the associated target genes. While SYNCRIP enhances SIRT1 expression through mRNA stabilization and transcriptional activation, SYNCRIP also regulates *HK2* expression through promoter interactions and IRES-mediated translation. This dual regulatory capacity enables SYNCRIP to support GBM progression by enhancing glycolytic activity and suppressing ferroptotic cell death, ultimately promoting tumorigenesis and metastasis. Collectively, our study establishes SYNCRIP as a central regulator that integrates transcriptional regulation, mRNA stability, and cap-independent translation to coordinate redox homeostasis and metabolic adaptation in GBM. These findings have important implications for overcoming treatment resistance in GBM and improving clinical outcomes.

## Materials and Methods

### Plasmid constructs

The lentiCRISPRv2 vector was used as the backbone to construct the plasmid for CRISPR-mediated silencing of SYNCRIP 50, 51. The vector was first digested with BsmBI (R0739L, NEB, USA) and dephosphorylated with CIP (M0525S, NEB) to facilitate the insertion of the SYNCRIP target sequence. Complementary oligonucleotides (5′-CACCGCCTGAATAAACGGAATCTGG-3′ and 5′-AAACCCAGATTCCGTTTATTCAGGC-3′) were annealed to form duplexes for construction of LC-SYNCRIP. After polynucleotide kinase treatment, the annealed oligonucleotides were ligated into the digested vector.

To construct the pRF *HK2* 5′ UTR plasmid, the 5′ UTR of mouse *HK2* was amplified using Pfu polymerase (SolGent). The PCR product was digested with SalI/SmaI and inserted into the intergenic region of the pRF bicistronic vector. To investigate the functional role of the R6 region in the *SIRT1* promoter, a series of luciferase reporter constructs was obtained from VectorBuilder (USA), including a promoterless basic vector, a construct containing the full-length human *SIRT1* promoter, including the R6 site, and promoter fragments corresponding to regions upstream and downstream of the R6 site that lack the R6 region (vector IDs: VB260105-1480rwj, VB260105-1503wsw, VB260105-1505cps, and VB260105-1513uay).

### Cell culture and treatment

LC-GFP or LC-SYNCRIP plasmids were transfected into cells using Lipofectamine 3000 (L3000015, Thermo Fisher Scientific, USA) according to the manufacturer's instructions to generate control or SYNCRIP-deficient (KO) cells. After 48 hours, screening was initiated using 1 µg/mL puromycin. The human GBM cell lines U373 (KCLB No. 30017), U87 (KCLB No. 30014), and HS683 (KCLB No. 30138) were obtained from the Korean Cell Line Bank (KCLB, Seoul, Republic of Korea). Puromycin-resistant cells were maintained in minimal essential medium (MEM; 11095-080, Gibco, USA) supplemented with 10% fetal bovine serum (FBS; 12483-020, Gibco), 1% penicillin-streptomycin, and 1 µg/mL puromycin (BML-GR312-0250, ENZO, USA), and cultured at 37°C in an incubator with 5% CO₂.

SYNCRIP-restored cells were generated by introducing EGFP-Mock or EGFP-SYNCRIP plasmids into the cells. Similarly, SIRT1-restored cells were generated by introducing Flag-Mock or Flag-SIRT1 plasmids into the cells. All transfections were performed using Lipofectamine 3000 according to the manufacturer's protocol. After 48 hours, the cells were collected and used for subsequent experiments.

To induce ferroptosis, cells were treated with various concentrations of erastin (Sigma-Aldrich, E7781), prepared from a of 5 mM stock solution for 72 hours. For ferroptosis inhibition experiments, ferrostatin-1 (Fer-1; SML0583, Sigma-Aldrich) was prepared as a 10 mM stock solution and applied to cells for 24 hours at the concentrations specified in the respective figure legends. To evaluate mRNA stability, transcription was inhibited in cells by treatment with actinomycin D (A9415, Sigma-Aldrich, USA) at a concentration of 10 µg/mL. Cells were harvested at 0, 2, 4, 6, and 8 hours after treatment.

### Xenograft animal model

U373 cells (1 × 10⁶ cells per mouse) were resuspended in serum-free medium and mixed with Matrigel (356234, Corning, USA) at a 1:1 (v/v) ratio immediately before injection. The cell suspension was injected subcutaneously into the lateral flank of each mouse. Tumor formation was monitored regularly after injection. Tumor size was measured every 2-5 days using digital calipers, and tumor volume was calculated using the formula 0.52 × length × width² based on an ellipsoid approximation. Tumor growth was monitored longitudinally throughout the experiment. The experiment was terminated on day 124 post-injection based on humane endpoint considerations, and mice were sacrificed at this time point for subsequent analyses.

To further assess the role of ferroptosis in tumor growth, an additional experiment was performed in which mice were subcutaneously implanted with LC-GFP and LC-SYNCRIP cells on opposite flanks. One week after tumor implantation, once tumors were established, mice were treated with either vehicle control (DMSO) or Fer-1 at 10 mg/kg. Treatment was administered via intraperitoneal injections three times per week.

### Dual luciferase reporter assay

For the reporter assay, cells were transfected with a Gaussia luciferase (GLUC) plasmid containing either the human *SIRT1* promoter (HRPM35661-PG02, Genecopoeia, USA) or the mouse *HK2* promoter (MPRM42494-PG02, Genecopoeia) sequence, along with a Renilla luciferase plasmid. For GLUC-based reporter assays, culture supernatants were collected, and GLUC activity was measured using the Gaussia Luciferase Assay kit (LF061, Genecopoeia) in accordance with the manufacturer's instructions. Renilla luciferase was used as an internal control to normalize transfection efficiency.

For luciferase reporter assays, cells were transfected with either a psiCHECK2 plasmid containing the human *SIRT1* 3′ UTR sequence (VectorBuilder) or a pRF plasmid containing the *HK2* 5′ UTR sequence. Following transfection, the cells were lysed with reporter lysis buffer (Promega), and luciferase activity was measured with the Dual-Luciferase® Reporter Assay System (Promega, E1910) according to the manufacturer's protocol and quantified using a GloMax® 20/20 Luminometer (Promega, USA). Renilla and firefly luciferase activities were quantified separately.

### Immunofluorescence

A tissue array (GL807a) containing 80 brain tumors and normal tissues was purchased from US Biomax (Rockville, MD). After deparaffinization and hydration, the sections were incubated in pepsin (Abcam, ab801437) for 10 minutes, and then in a 3% hydrogen peroxide solution for an additional 10 minutes. After blocking with 2% bovine serum albumin (BSA) at room temperature for 1 hour, the sections were incubated with primary antibody against SYNCRIP (R5653, Sigma-Aldrich) at 4 ˚C for 1 day. The next day, the sections were incubated with Fluor® 647-conjugated rabbit secondary antibody (Abcam, ab150067) at room temperature for 1 hour. Fluorescence microscopy images were obtained with an EVOS FL Auto Imaging System (Thermo Fisher Scientific, USA), and fluorescence image intensity was analyzed with ImageJ software.

To evaluate DNA synthesis and cell proliferation, 5-bromo-2'-deoxyuridine (BrdU) incorporation assays were performed. Cells were incubated with 10 µM BrdU for 30 minutes under standard culture conditions. After labeling, cells were fixed, permeabilized, and treated with 2 N HCl at room temperature for 30 minutes to denature the DNA, then neutralized with 0.1 M sodium borate (pH 8.5). After blocking, cells were incubated with an anti-BrdU antibody (Thermo Fisher Scientific, MA3-071), followed by incubation with a Fluor® 488-conjugated secondary antibody (Abcam, ab150117). Nuclei were counterstained with Hoechst 33342, and images were acquired using the EVOS FL Auto Imaging System. BrdU-positive cells were quantified using ImageJ software by calculating the percentage of BrdU-positive nuclei relative to total nuclei.

### Proliferation assay

For cell counting analysis, control and SYNCRIP-deficient cells were seeded at 2000 cells/well in a 24-well plate. The medium was removed every 24 hours, and 200 µL of TrypLE™ solution (Gibco, 12605-028) was added to dissociate the cells for cell counting.

For the colony formation assay, U373, U87, and HS683 cells were seeded at 500 cells per well in 6-well plates. Cells were cultured for 14 days to allow colony formation. After incubation, the wells were gently washed twice with phosphate-buffered saline (PBS), and the cells were fixed with 4% paraformaldehyde at room temperature for 15 minutes. The fixed cells were then stained with 0.1% crystal violet at room temperature for 30 minutes, followed by washing with distilled water to remove excess dye. Images of the colonies were captured using a digital camera.

### Migration and invasion assay

For the spheroid 3D invasion assay, U373 and U87 cells were seeded into round-bottom 96-well plates and cultured for 2 days to allow spheroid formation. Once spheroids had formed, the medium was replaced, and 50 μL of Matrigel (Corning, 354230) was added to each well to establish a semi-solid matrix. After 2 hours of solidification, 100 µL of fresh medium was added to prevent drying. Invasive cell migration was monitored using phase-contrast microscopy for 9 days (U373) or 5 days (U87), and invasion was quantified by measuring extensions from the spheroid surface using ImageJ.

For the wound healing assay, 2 × 10⁵ cells were seeded into 12-well plates and cultured for 24 hours. A linear scratch was introduced using a 200 µL pipette tip, followed by washing with PBS to remove detached cells. Fresh culture medium was then added, and wound closure was monitored over time.

For the migration assay, a 24-well Transwell insert system with an 8.0 µm-pore size polycarbonate membrane (Corning, Inc.) was used. U373 and HS683 cells (5 × 10⁴) or U87 cells (3 × 10⁴) were suspended in 300 µL of serum-free MEM and seeded into the upper chamber, while 750 µL of MEM containing 10% FBS was added to the lower chamber as a chemoattractant. After 24 hours of incubation at 37 °C, cells were washed with PBS, fixed with 4% paraformaldehyde, and stained with 0.1% crystal violet at room temperature for 20 minutes. Non-migrated cells were removed from the upper membrane surface using a cotton swab. Images were acquired using the EVOS FL Auto Imaging System (Thermo Fisher Scientific) at 20× magnification.

### RNA extraction and quantitative real-time PCR (RT-qPCR)

Total RNA was isolated from each GBM cell line using the Blood/Cultured Cell Total RNA Mini kit (FABRK001-1, Favorgen Biotech, Taiwan) according to the manufacturer's instructions. The extracted RNA was treated with RNase-free DNase to remove residual genomic DNA contamination and then inactivated. Purified RNA was reverse-transcribed into cDNA using the First Strand cDNA Synthesis kit (K1622, Thermo Fisher Scientific, USA) according to the manufacturer's protocol.

Quantitative real-time PCR (RT-PCR) was performed using Power SYBR Green Master Mix under standard amplification conditions. Relative mRNA expression levels were calculated using the 2^-ΔΔCt method. All primer sequences used in this study are listed in Table S1.

### Immunoblot analysis

Cells were directly lysed in Laemmli buffer (60 mM Tris-HCl (pH 6.8), 2% (w/v) sodium dodecyl sulfate (SDS), 10% (v/v) glycerol, and 0.02% (w/v) bromophenol blue), then sonicated and denatured by boiling at 95 ℃ for 5 minutes. Protein samples were separated via SDS-polyacrylamide gel electrophoresis (SDS-PAGE) using a 12% gel and transferred to a polyvinylidene fluoride (PVDF) membrane. After blocking with 5% skimmed milk at room temperature for 1 hour, the membrane was incubated overnight at 4 °C with the following primary antibodies (1:1000 dilution unless otherwise indicated): anti-transferrin receptor (TFRC; 13-6800, Thermo Fisher Scientific), anti-SYNCRIP (R5653, Sigma-Aldrich), anti-SIRT1 (ab110304, Abcam, UK), anti-NRF2 (NBP1-32822, Novus, USA), anti-xCT (ab37185, Abcam), anti-GPX4 (ab125066, Abcam), anti-GFP (2956, Cell Signaling, USA), anti-Cleaved caspase 3 (9661, Cell Signaling), anti-PRDX3 (A304-744A, Bethyl Laboratories, USA), anti-PRDX5 (A305-339A, Bethyl Laboratories), anti-HK2 (ab209847, Abcam), and anti-ACTIN (A5316, Sigma- Aldrich, USA). The next day, the membrane was incubated with horseradish peroxidase-conjugated secondary anti-rabbit (1:10,000, Abcam, ab205718) and anti-mouse (1:10,000, Bethyl Laboratories, A90-116P) antibodies at room temperature for 1 hour. Immune signals were detected using the D-Plus™ ECL Femto system (ECL-FS200, Donginbiotech Co.). Quantification of Western blots was performed using ImageJ.

### RNA immunoprecipitation (IP-RNA)

IP-RNA was performed using the Magna RIP™ RBP Immunoprecipitation kit (17-700, Millipore, USA) according to the manufacturer's instructions.

Briefly, cells were lysed using the RIP lysis buffer provided in the kit, and cell lysates were incubated with magnetic beads pre-coated with either anti-SYNCRIP antibody (R5653, Sigma-Aldrich) or purified mouse IgG (5415, Cell Signaling Technology) as a negative control. Immunoprecipitation was performed overnight at 4 °C with gentle rotation. After extensive washing to remove nonspecifically bound materials, RNA-protein complexes were digested with proteinase K to release the immunoprecipitated RNA. The recovered RNA was purified and reverse-transcribed into cDNA, followed by RT-qPCR analysis using a real-time PCR system (MIC-2, Bio Molecular Systems, Australia). Enrichment of human *SIRT1* mRNA in SYNCRIP immunoprecipitates was assessed by RT-qPCR, and the primer sequences used for amplification are listed in Table S1.

### MDA and GSH/GSSG assays

Cells were seeded in 100 mm culture dishes at a density of 8 × 10⁵ cells per dish and incubated at 37 °C for 24 h. Subsequently, cells were treated with erastin (10 µM) for an additional 24 h. For ferroptosis inhibition experiments, U373 and HS683 cells were treated with Fer-1 (50 µM) for 24 h. Following treatment, cells were washed twice with ice-cold PBS, collected by centrifugation at 7000 rpm for 2 min, and processed for subsequent analyses. Lipid peroxidation was assessed by measuring MDA levels using a Lipid Peroxidation Assay kit (ab118970, Abcam) according to the manufacturer's instructions, with absorbance measured at 532 nm on a microplate reader (Tecan, Switzerland). Intracellular GSH and GSSG levels were quantified using the GSH/GSSG-Glo™ Assay kit (V6612, Promega, USA) following the manufacturer's protocol, and luminescence was measured using a GloMax® 20/20 Luminometer (Promega, USA).

### ROS and mitochondrial membrane potential analyses

Intracellular ROS levels were measured using CellROX (C10443, Thermo Fisher Scientific) according to the manufacturer's instructions. Mitochondrial membrane potential was assessed using the TMRE Mitochondrial Membrane Potential Assay kit (ab113852, Abcam). Cells were incubated with the respective fluorescent probes in the dark at 37°C for 30 min and then washed with PBS. For nuclear counterstaining, cells were incubated with Hoechst 33342 (R37605, Invitrogen, USA) at room temperature for 10 min. Fluorescence images were acquired using the EVOS FL Auto Imaging System (Thermo Fisher Scientific). Fluorescence intensity was quantified using ImageJ software (National Institutes of Health).

### Chromatin Immunoprecipitation (ChIP) assay

For cell cross-linking, cells were treated with 1% formaldehyde at room temperature for 1 hour, and cross-linking was stopped by incubation with 200 mM glycine for 5 min. Cells were then lysed with lysis buffer (1% SDS, 10 mM EDTA, 50 mM Tris [pH 8.0]). The lysate was sonicated and mixed with dilution buffer (0.01% SDS, 1.1% Triton X-100, 1.2 mM EDTA, 16.7 mM Tris-HCl [pH 8.0], 150 mM NaCl). The denatured DNA was incubated with SYNCRIP antibody (Sigma-Aldrich, R5653) and mouse IgG antibody (Cell Signaling, 5415) at 4 °C overnight. The solution was then mixed with Dynabeads Protein G (Thermo Fisher Scientific, 10003D) for 3 hours. Beads were separated from the solution, and bound complexes were eluted using SDS-carbonate buffer (1% SDS and 0.1 M NaHCO₃). For reversal of cross-links, the eluted samples were mixed with 5 M NaCl and incubated overnight at 65 °C. Samples were then treated with RNase A (Roche) and Proteinase K (Roche), and the DNA was purified using the QIAquick PCR Purification kit (QIAGEN, 28106) according to the manufacturer's instructions. Enriched DNA fragments were analyzed by qPCR using primers specific for the *SIRT1* promoter region.

### Oxygen Consumption Rate (OCR)

Extracellular flux analysis was conducted using the Seahorse XF Cell Mito Stress Test Kit (Seahorse Bioscience, 103010-100) following the manufacturer's protocol. Cells were seeded at 1.5 × 10⁴ cells per well in Seahorse XF plates and maintained for 24 hours. At the time of the assay, the culture medium was replaced with Seahorse XF assay medium supplemented with glucose and glutamine, and cells were equilibrated for 1 hour in a non-CO₂ incubator. OCR was measured using a Seahorse XF analyzer (Seahorse Bioscience, Billerica, MA, USA). Baseline respiration was recorded first, followed by injection of oligomycin (1.5 µM), FCCP (1.5 µM), and rotenone/antimycin A (0.5 µM) to assess mitochondrial function. OCR values were normalized to cell number per well, determined by cell counting after the assay, and analyzed using Wave software.

### Annexin V/ propidium iodide (PI) apoptosis assay

Apoptosis was evaluated using the eBioscience Annexin V-FITC Apoptosis Detection kit (BMS500FI-20, Thermo Fisher Scientific) according to the manufacturer's instructions. U373, U87, and HS683 cells were prepared at 2 × 10^5^ cells per well, washed with PBS, and resuspended in 1× binding buffer. Cells were then stained with FITC-Annexin V and PI in the dark at room temperature for 10-15 min. The stained cells were analyzed promptly using a BD FACSAria Fusion flow cytometer (BD Biosciences, USA). FITC and PI fluorescence signals were measured, with unstained cells used as negative controls for gating. Data were analyzed using FlowJo software.

### Statistical analysis

Statistical analyses were performed using GraphPad Prism version 8.4 (GraphPad Software, Inc.). Data were analyzed using a two-tailed Student's t-test, one-way analysis of variance (ANOVA), or two-way ANOVA, followed by Dunnett's or Šidák's multiple comparison test. A *P*-value of less than 0.05 was considered statistically significant (*P < 0.05, **P< 0.01, and ***P < 0.001).

## Supplementary Material

Supplementary figures and table.

## Data Availability

The data supporting the results of this study are presented in the article and in the online supplementary information file.

## Abbreviations

SYNCRIP: synaptotagmin-binding cytoplasmic RNA-interacting protein
hnRNP Q: heterogeneous nuclear ribonucleoprotein Q
RBP: RNA-binding protein
GBM: glioblastoma
ROS: reactive oxygen species
IRES: internal ribosome entry site
HK2: hexokinase 2
EMT: epithelial-mesenchymal transition
GPX4: glutathione peroxidase 4
GSH: glutathione
SIRT1: sirtuin 1
NRF2: nuclear factor erythroid 2-related factor 2
GEPIA: Gene Expression Profiling Interactive Analysis
NHAs: normal human astrocytes
GCLC: glutamate-cysteine ligase catalytic subunit
NQO1: NAD(P)H quinone dehydrogenase 1
MDA: malondialdehyde
TFRC: transferrin receptor
ActD: actinomycin D
3' UTR: 3' untranslated region
TMRE: tetramethylrhodamine ethyl ester
OCR: oxygen consumption rate
HuR: Hu-antigen R
Fer-1: ferrostatin-1
