# Supplementary material for: SYNCRIP drives ferroptosis resistance and metabolic activation via SIRT1 and HK2 in glioblastoma
Source: Int J Biol Sci. 2026 Jul 13;22(12):6670–88. doi: 10.7150/ijbs.127096 (PMC13412200; doi:10.7150/ijbs.127096)
Supplement: Supplementary file 1 — Supplementary figures and table. [file ijbsv22p6670s1.pdf]

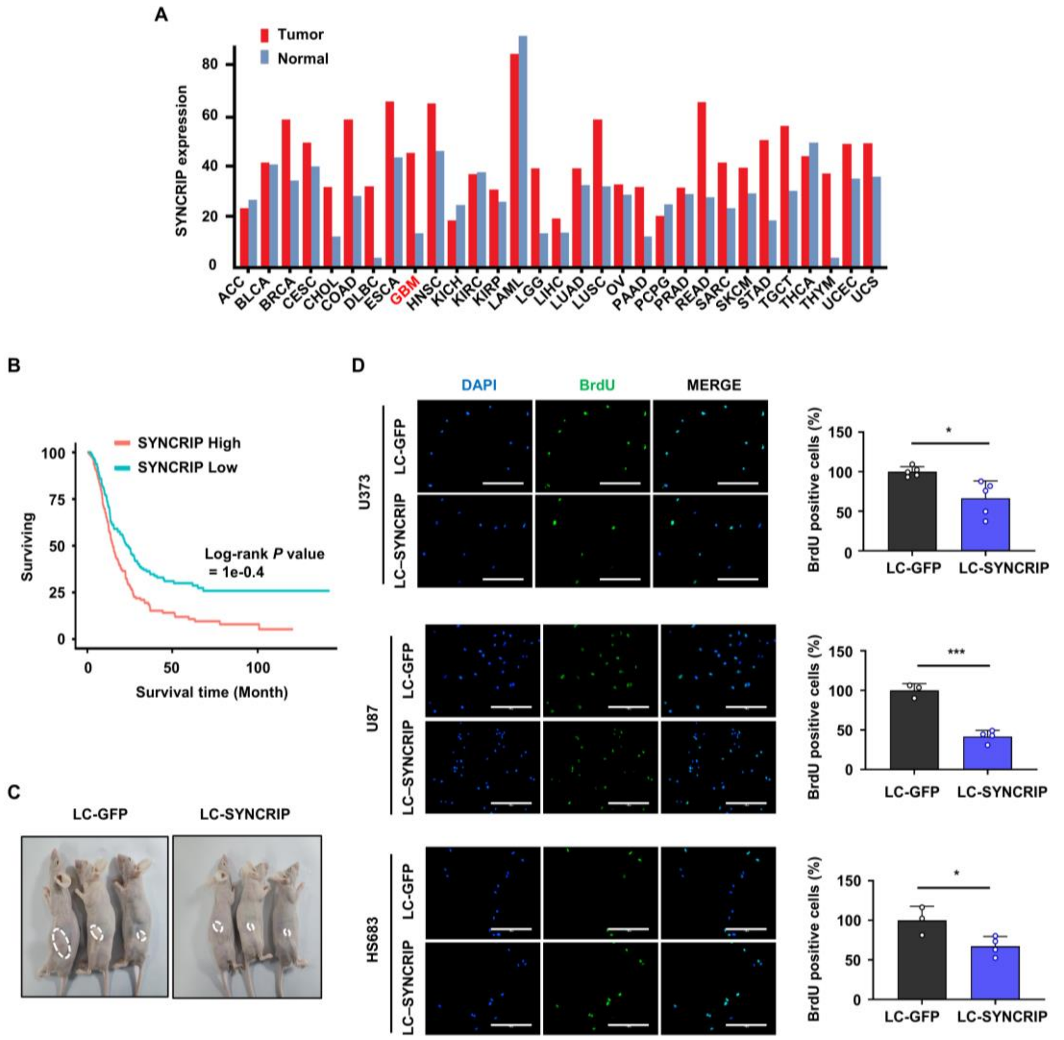
A
Tumor
Normal
SYNCRIP expression
ACC BLCA BRCA CESC CHOL COAD DLBC ESCA GBM HNSC KICH KIRC KIRP LAML LGG LIHC LUAD LUSC OV PAAD PCPG PRAD READ SARC SKCM STAD TGCT THCA THYM UCEC UCS
B
SYNCRIP High
SYNCRIP Low
Surviving
Log-rank P value = 1e-0.4
Survival time (Month)
C
LC-GFP
LC-SYNCRIP
D
DAPI
BrdU
MERGE
U373
U87
HS683
LC-GFP
LC-SYNCRIP
BrdU positive cells (%)
LC-GFP
LC-SYNCRIP

Figure S1. Expression of SYNCRIP is associated with poor prognosis and increased proliferation in GBM. A. The Gene Expression Profiling Interactive Analysis (GEPIA) database-derived expression profile shows elevated SYNCRIP expression across various tumor types compared with normal tissues. **B**. Kaplan–Meier survival analysis shows that high SYNCRIP expression is significantly associated with poorer overall survival among patients with GBM. **C**. Representative tumor images from xenograft experiments are shown. Tumors derived from LC-GFP and LC-SYNCRIP cells are presented. These images were selected from the same cohort of mice (n = 5 per group) used for the quantitative analyses of tumor number and tumor volume and are provided for visualization purposes. The images are consistent with the overall trends observed across all animals. **D**. The 5-bromo-2'-deoxyuridine (BrdU) analysis shows reduced proliferation in SYNCRIP-deficient cells. Representative images of BrdU staining (left) and quantification of BrdU-positive cells (right) are shown for U373 (top; n = 5), U87 (middle; n = 4) and HS683 (bottom; n = 3). Scale bar, 200 μm. Student's t-test was used to assess statistical significance (*, $P < 0.05$; ***, $P < 0.001$.). Data are presented as the mean ± standard error of the mean (SEM).

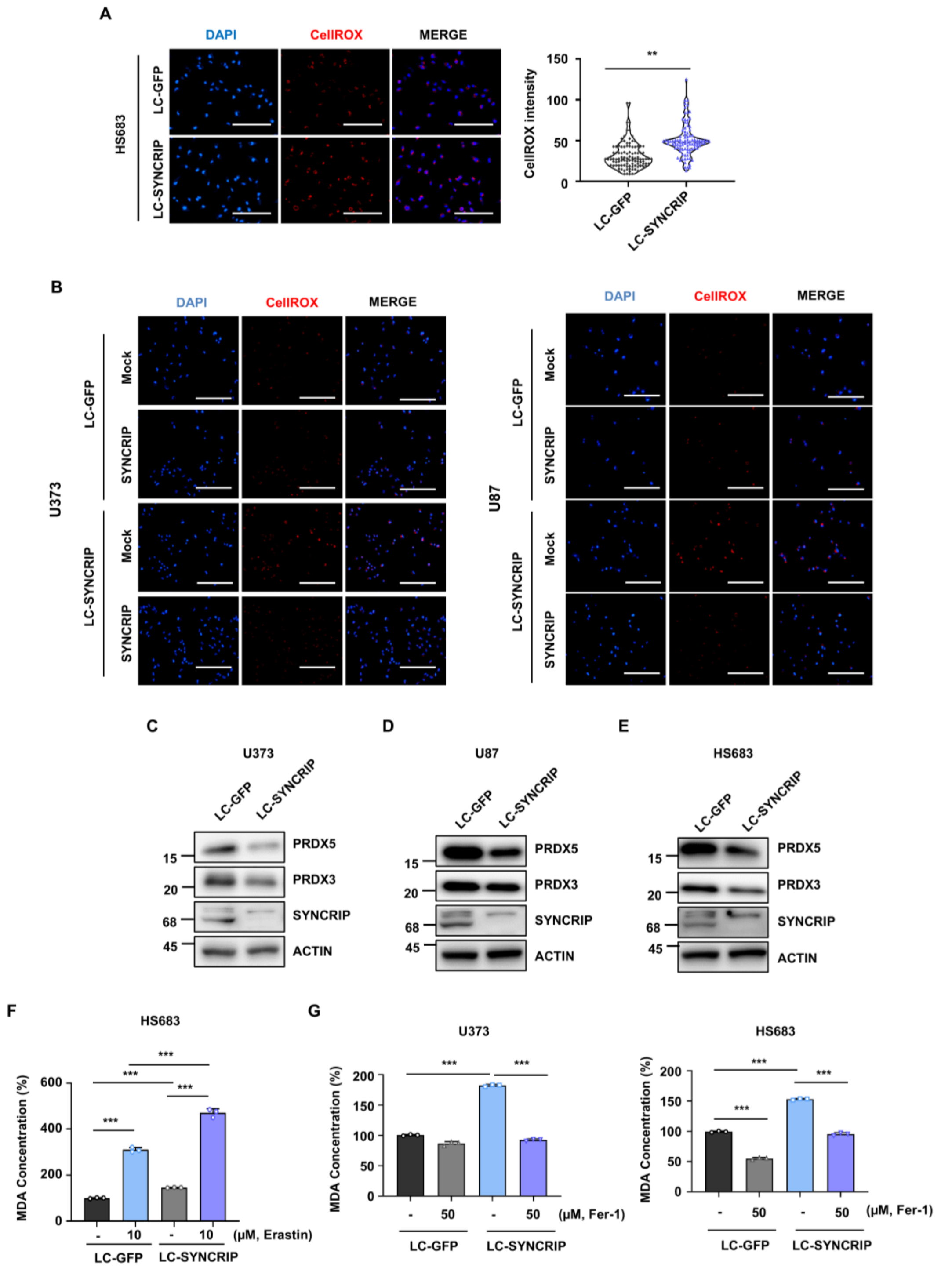
A
DAPI
CellROX
MERGE
HS683
LC-GFP
LC-SYNCRIP
CellROX intensity
150
100
50
0
**
LC-GFP
LC-SYNCRIP
B
DAPI
CellROX
MERGE
U373
LC-GFP
LC-SYNCRIP
Mock
SYNCRIP
U87
C
U373
D
U87
E
HS683
LC-GFP
LC-SYNCRIP
PRDX5
PRDX3
SYNCRIP
ACTIN
15
20
68
45
F
HS683
MDA Concentration (%)
600
400
200
0
***
(μM, Erastin)
G
U373
HS683
200
150
100
50
(μM, Fer-1)

Figure S2. SYNCRIP depletion increases ROS accumulation. A. Representative images (left) and quantification (right) of ROS levels in control and SYNCRIP-deficient HS683 cells, showing a significant increase in ROS accumulation upon SYNCRIP depletion. Scale bar, 200 μm. Student's t-test was used to assess statistical significance. **B**. CellROX staining images show decreased ROS levels after SYNCRIP reintroduction in SYNCRIP-KO cells. Scale bar, 200 μm. **C–E**. Western blot analysis of antioxidant-related protein expression, including PRDX3, PRDX5, and SYNCRIP, in SYNCRIP-deficient and control cells. ACTIN was used as a loading control. **F**. MDA levels were measured in HS683 cells treated with increasing concentrations of erastin, which show enhanced lipid peroxidation upon SYNCRIP depletion. **G**. MDA levels were measured in U373 (left) and HS683 (right) cells after treatment with DMSO or ferrostatin-1 (Fer-1; 50 μM) for 24 h. Fer-1 treatment significantly reduced MDA levels. Data are presented as the mean ± SD. Statistical significance was determined using one-way analysis of variance (ANOVA) with Dunnett's post hoc test (***, $P < 0.001$).

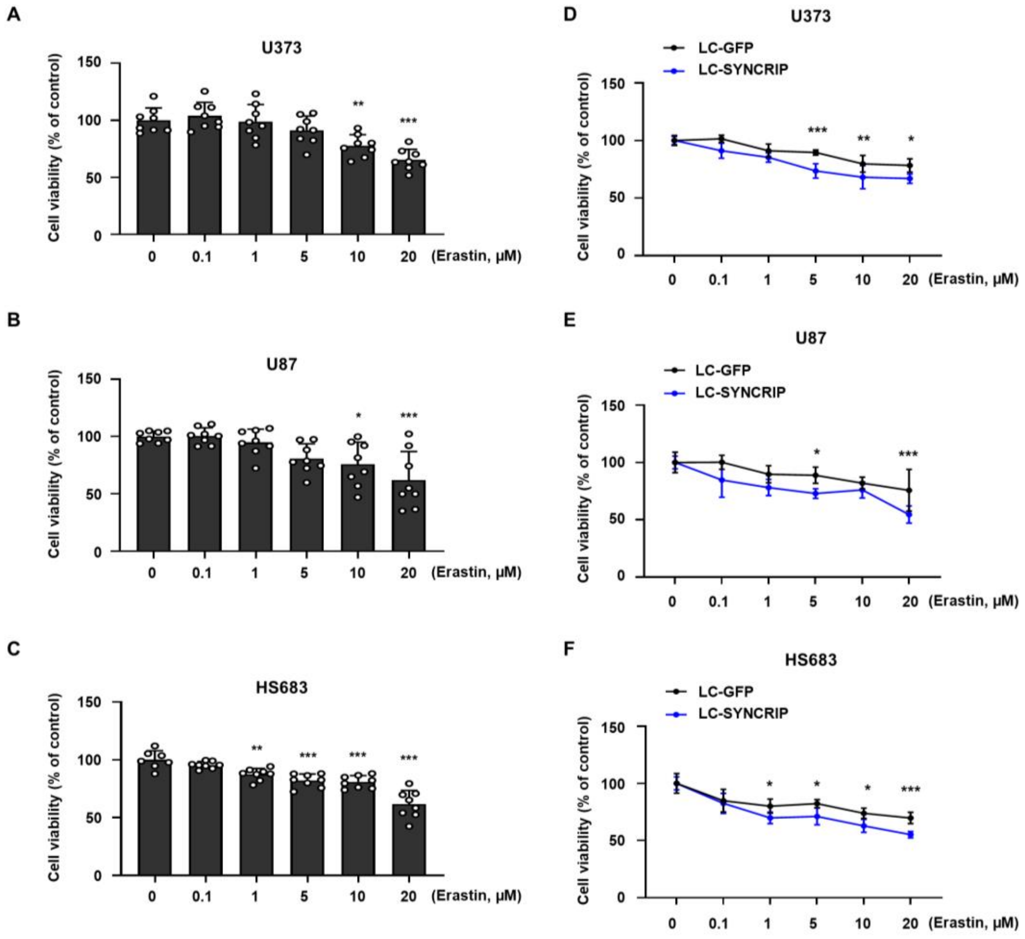
A
U373
Cell viability (% of control)
150
100
50
0
**
***
0
0.1
1
5
10
20
(Erastin, μM)
B
U87
*
***
C
HS683
**
***
***
***
D
U373
LC-GFP
LC-SYNCRIP
***
**
*
E
U87
*
***
F
HS683
*
*
*
***

Figure S3. SYNCRIP deficiency increases susceptibility to erastin-induced ferroptosis. A–C. CCK assay analysis of erastin induced ferroptosis in various GBM cell lines. U373 (**A**), U87 (**B**), and HS683 (**C**). One-way ANOVA with Dunnett's post hoc test was used (n = 8, *, $P < 0.05$; **, $P < 0.01$; ***, $P < 0.001$). **D–F**. CCK assay analysis of cell viability in control and SYNCRIP-deficient U373 (**D**), U87 (**E**), and HS683 (**F**) cells after dose-dependent erastin treatment. Two-way ANOVA with Šidák's multiple comparisons was used (n = 8, *, $P < 0.05$; **, $P < 0.01$; ***, $P < 0.001$). Data are presented as the mean ± SD.

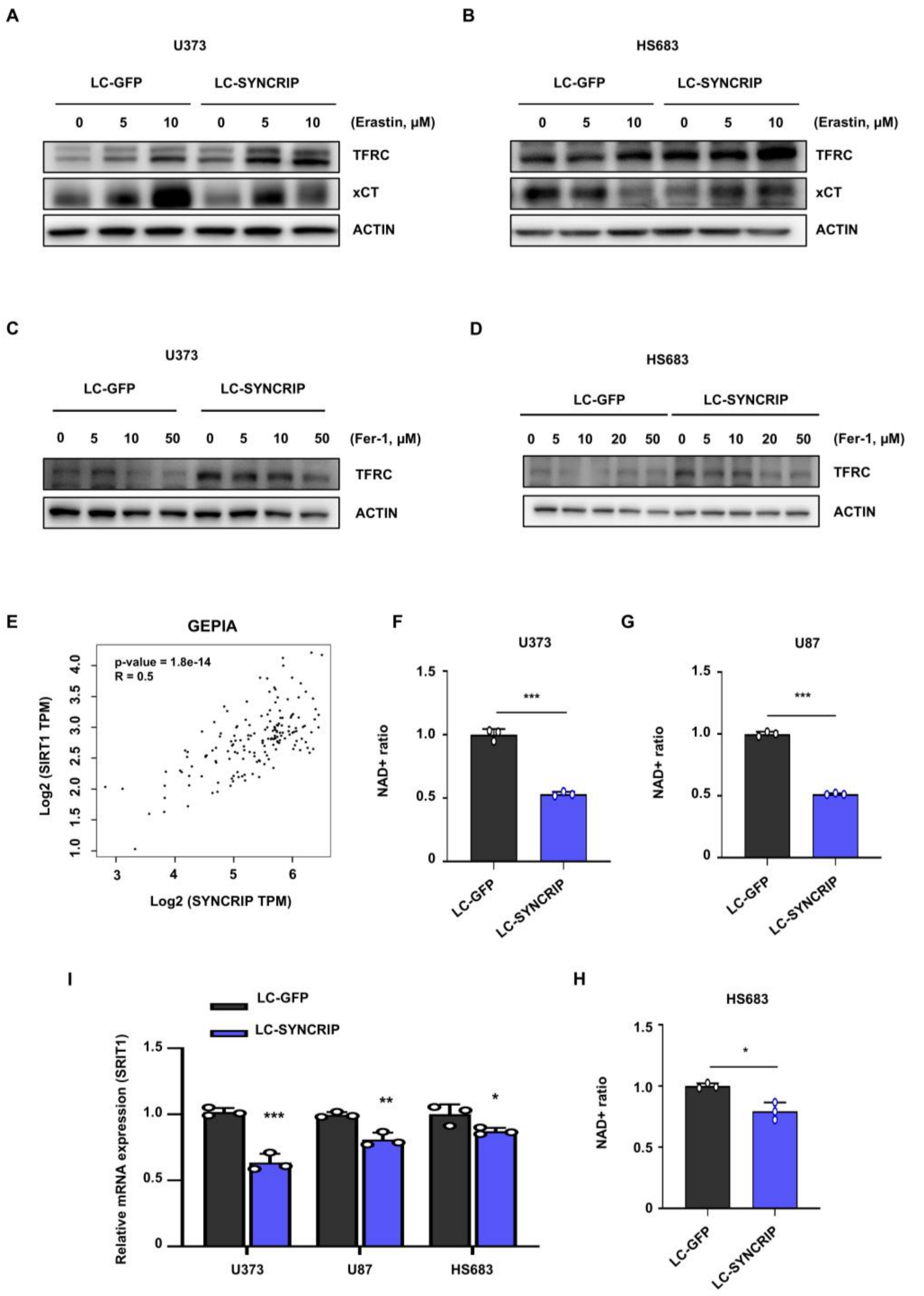
A
U373
LC-GFP
LC-SYNCRIP
0 5 10 0 5 10 (Erastin, μM)
TFRC
xCT
ACTIN
B
HS683
LC-GFP
LC-SYNCRIP
0 5 10 0 5 10 (Erastin, μM)
TFRC
xCT
ACTIN
C
U373
LC-GFP
LC-SYNCRIP
0 5 10 50 0 5 10 50 (Fer-1, μM)
TFRC
ACTIN
D
HS683
LC-GFP
LC-SYNCRIP
0 5 10 20 50 0 5 10 20 50 (Fer-1, μM)
TFRC
ACTIN
E
GEPIA
p-value = 1.8e-14
R = 0.5
Log2 (SIRT1 TPM)
Log2 (SYNCRIP TPM)
F
U373
NAD+ ratio
***
LC-GFP
LC-SYNCRIP
G
U87
NAD+ ratio
***
LC-GFP
LC-SYNCRIP
I
LC-GFP
LC-SYNCRIP
Relative mRNA expression (SRIT1)
***
**
*
U373
U87
HS683
H
HS683
NAD+ ratio
*
LC-GFP
LC-SYNCRIP

Figure S4. SYNCRIP attenuates ferroptosis through modulation of SIRT1 expression. A–B. Immunoblot analysis shows dose-dependent increases in transferrin receptor (TFRC) expression in SYNCRIP-deficient U373 (**A**) and HS683 (**B**) cells after erastin treatment. **C–D.** Immunoblot analysis shows decreased TFRC expression in SYNCRIP-deficient U373 (**C**) and HS683 (**D**) cells after dose-dependent treatment with ferrostatin-1 (Fer-1). **E**. GEPIA demonstrates a positive correlation between SYNCRIP and SIRT1 expression. **F–H**. NAD^+ assay shows a significant reduction in intracellular NAD^+ levels upon SYNCRIP depletion in U373 (**F**), U87 (**G**), and HS683 (**H**) cells. Student's t-test was used to assess statistical significance (n = 3, *, $P < 0.05$; ***, $P < 0.001$). **I**. RT-PCR analysis shows decreased *SIRT1* mRNA levels following SYNCRIP depletion. Two-way ANOVA with Šidák's multiple comparisons was used (n = 3, *, $P < 0.05$; **, $P < 0.01$; ***, $P < 0.001$.). All data are presented as the mean ± SD.

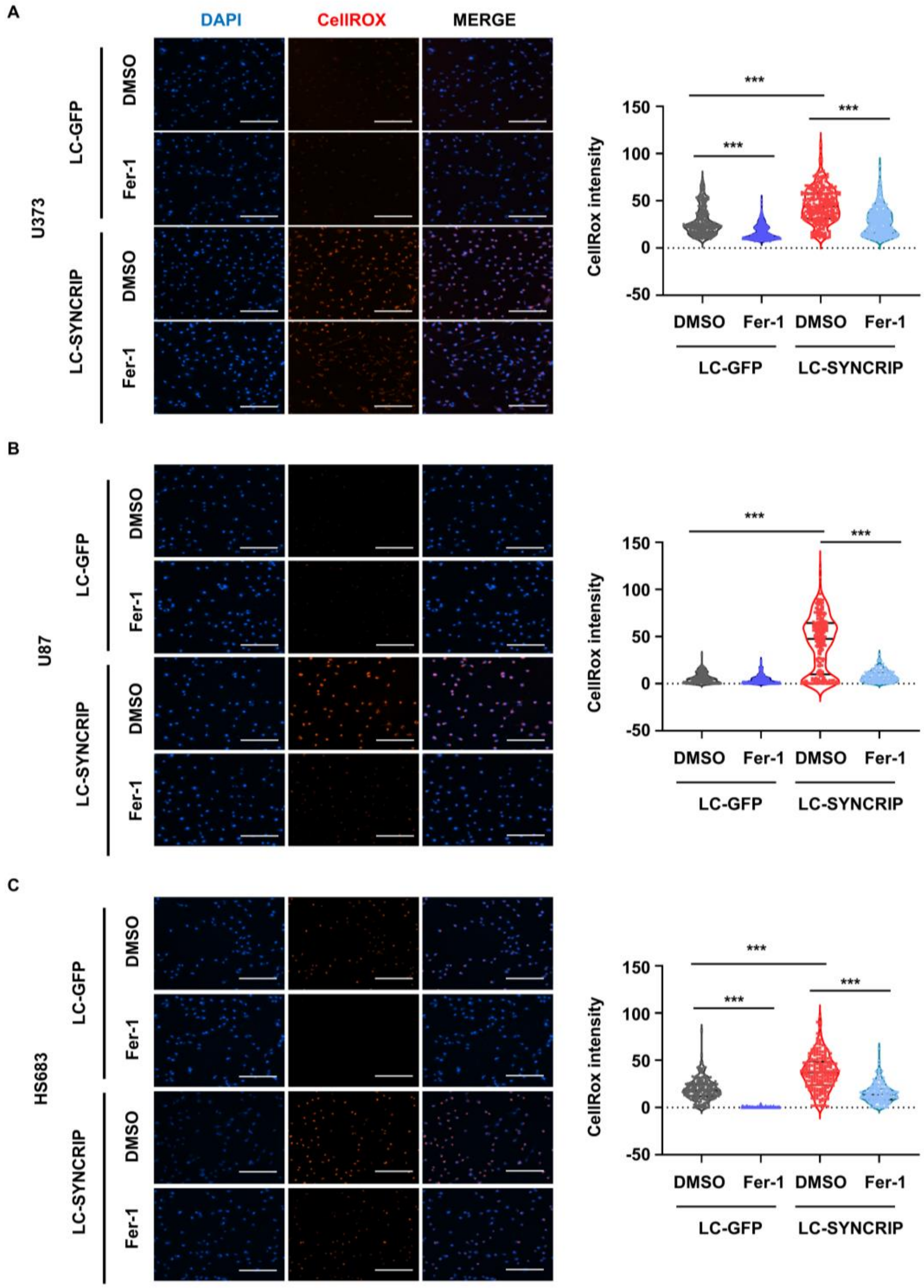
A
DAPI
CellROX
MERGE
U373
LC-GFP
LC-SYNCRIP
DMSO
Fer-1
CellRox intensity
***
B
U87
C
HS683

Figure S5. Ferrostatin-1 reduces ROS accumulation in SYNCRIP-deficient GBM cells. A–C. Representative images (left) and quantification (right) of CellROX fluorescence in U373 (**A,** n = 400–480), U87 (**B,** n = 150–200), and HS683 (**C,** n = 200–300) cells treated with DMSO or ferrostatin-1 (Fer-1, 50 μM). SYNCRIP-deficient cells exhibited increased ROS levels, which were significantly reduced upon Fer-1 treatment. Scale bar, 200 μm. Statistical significance was determined using one-way ANOVA with Dunnett's post hoc test (***, P < 0.001). Data are presented as the mean ± SD.

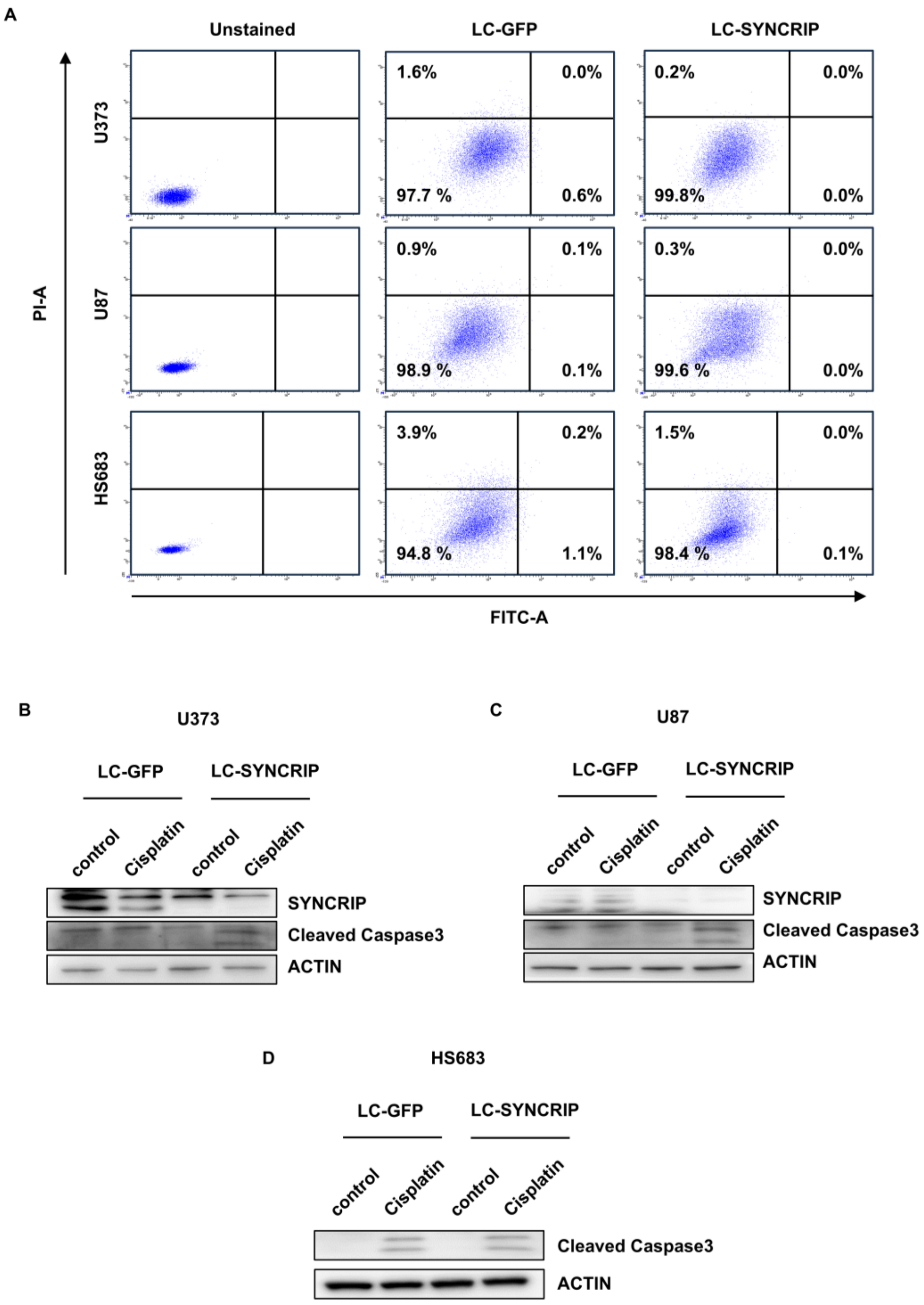
A
Unstained
LC-GFP
LC-SYNCRIP
PI-A
U373
U87
HS683
FITC-A
1.6%
0.0%
97.7 %
0.6%
0.2%
0.0%
99.8%
0.0%
0.9%
0.1%
98.9 %
0.1%
0.3%
0.0%
99.6 %
0.0%
3.9%
0.2%
94.8 %
1.1%
1.5%
0.0%
98.4 %
0.1%
B
U373
C
U87
LC-GFP
LC-SYNCRIP
control
Cisplatin
SYNCRIP
Cleaved Caspase3
ACTIN
D
HS683

Figure S6. SYNCRIP depletion does not induce apoptosis in glioblastoma cells. A. Annexin V/PI staining was used to analyze apoptotic and necrotic cell populations in U373, U87, and HS683 cells after SYNCRIP depletion. Representative flow cytometry plots are shown. **B–D.** Apoptosis was induced by treatment with cisplatin (Cayman Chemical, #13119) for 24 h in U373 and U87 cells at 10 μM and in HS683 cells at 20 μM. Cleaved caspase-3 expression was analyzed by Western blotting. ACTIN was used as the loading control.

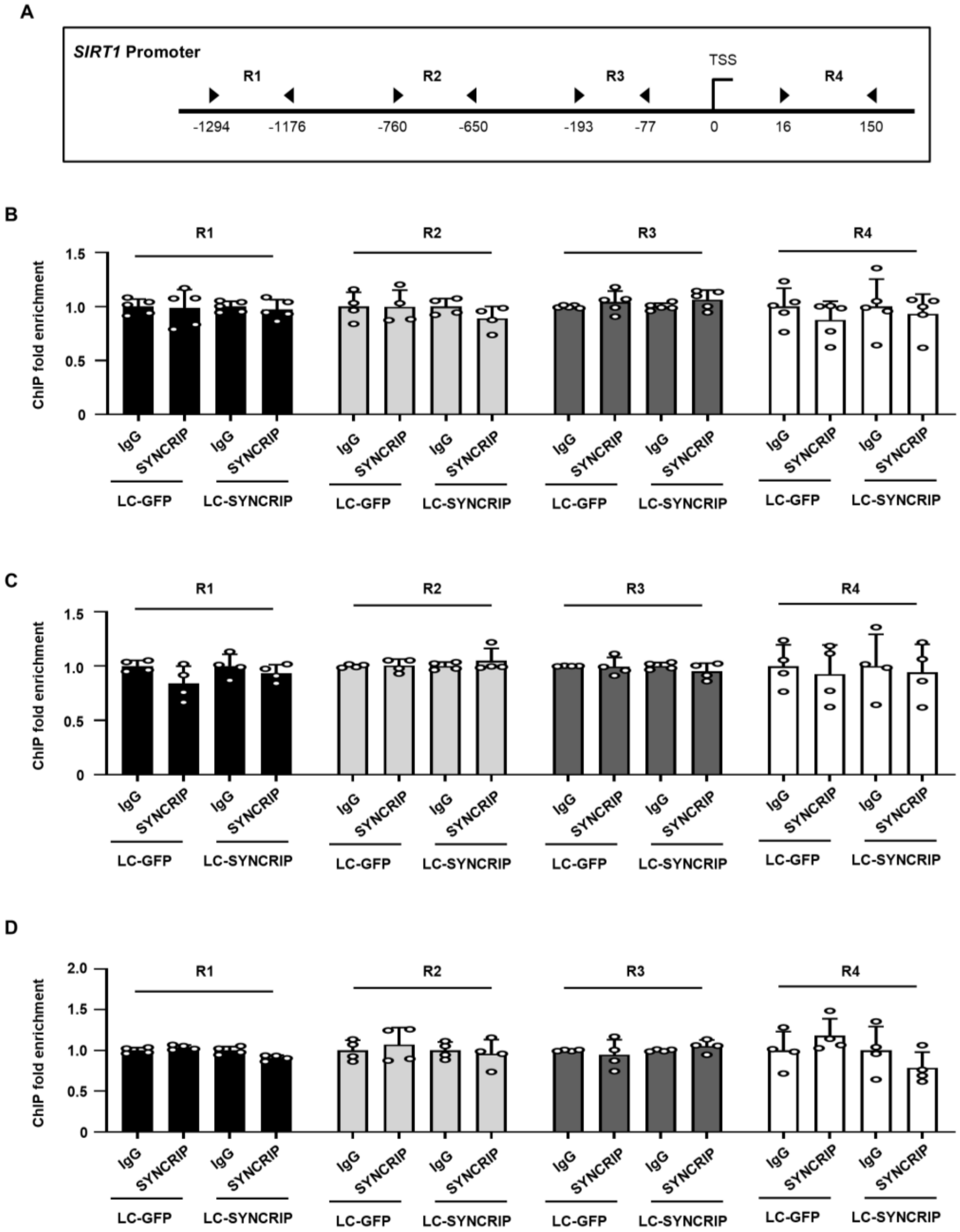
A
SIRT1 Promoter
R1
R2
R3
R4
TSS
-1294
-1176
-760
-650
-193
-77
0
16
150
B
C
D
ChIP fold enrichment
IgG
SYNCRIP
LC-GFP
LC-SYNCRIP

Figure S7. SYNCRIP specifically binds to the *SIRT1* promoter. A. Schematic representation of the *SIRT1* promoter region, indicating R1–R4. **B–D**. Chromatin immunoprecipitation (ChIP) analysis of SYNCRIP binding at the R1–R4 regions of the *SIRT1* promoter, showed no notable increase in binding affinity detected in these regions. U373 (**B**), U87 (**C**), and HS683 (**D**). Two-way ANOVA with Šidák's multiple comparisons was used.

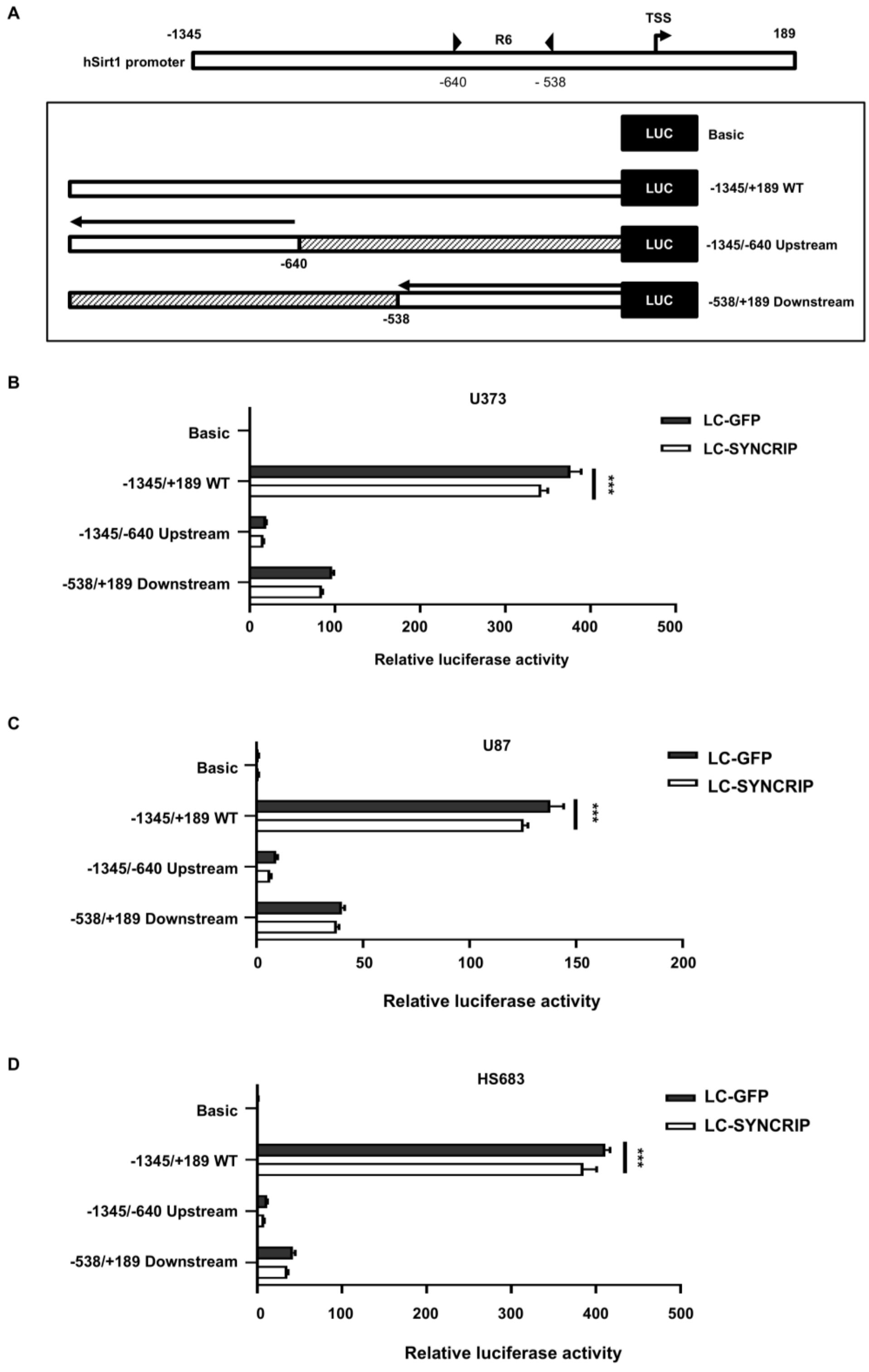
A
-1345
R6
TSS
189
hSirt1 promoter
-640
- 538
LUC
Basic
-1345/+189 WT
-1345/-640 Upstream
-640
-538/+189 Downstream
-538
B
U373
LC-GFP
LC-SYNCRIP
Basic
-1345/+189 WT
-1345/-640 Upstream
-538/+189 Downstream
***
0
100
200
300
400
500
Relative luciferase activity
C
U87
LC-GFP
LC-SYNCRIP
Basic
-1345/+189 WT
-1345/-640 Upstream
-538/+189 Downstream
***
0
50
100
150
200
Relative luciferase activity
D
HS683
LC-GFP
LC-SYNCRIP
Basic
-1345/+189 WT
-1345/-640 Upstream
-538/+189 Downstream
***
0
100
200
300
400
500
Relative luciferase activity

Figure S8. R6-dependent regulation of *SIRT1* promoter activity by SYNCRIP. A. Schematic illustration of SIRT1 promoter constructs. **B–D**. Luciferase reporter assays in U373 (**B**), U87 (**C**), and HS683 (**D**) cells transfected with the indicated constructs show R6-dependent regulation of SIRT1 promoter activity. Data are presented as the mean ± SD. Two-way ANOVA with Šidák's multiple comparisons was used (n = 3, ***, $P < 0.001$).

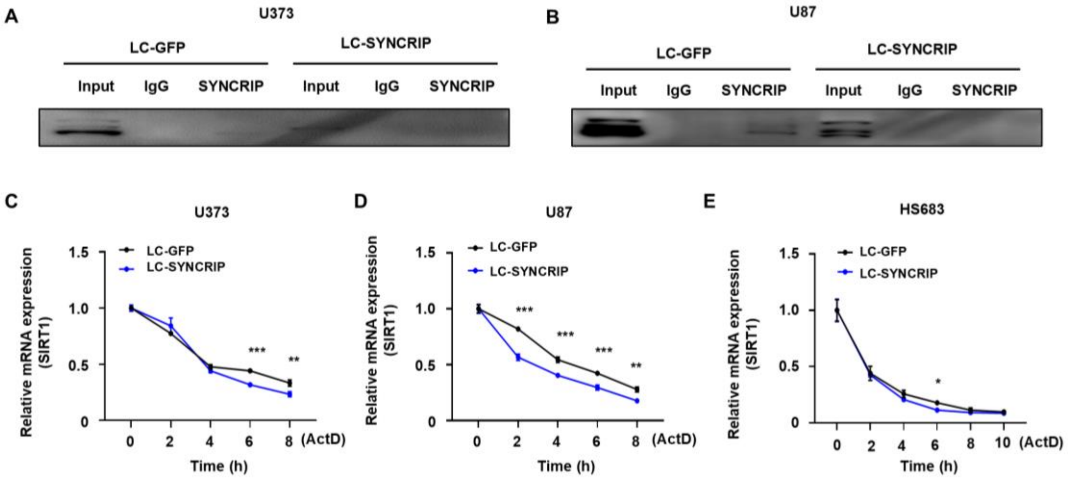
A
U373
LC-GFP
LC-SYNCRIP
Input
IgG
SYNCRIP
Input
IgG
SYNCRIP
B
U87
LC-GFP
LC-SYNCRIP
Input
IgG
SYNCRIP
Input
IgG
SYNCRIP
C
U373
LC-GFP
LC-SYNCRIP
Relative mRNA expression (SIRT1)
Time (h)
(ActD)
D
U87
LC-GFP
LC-SYNCRIP
Relative mRNA expression (SIRT1)
Time (h)
(ActD)
E
HS683
LC-GFP
LC-SYNCRIP
Relative mRNA expression (SIRT1)
Time (h)
(ActD)

Figure S9. SYNCRIP promotes the stabilization of *SIRT1* mRNA. A–B. Western blot analysis of SYNCRIP after IP-RNA in U373 (**A**) and U87 (**B**) cells confirmed SYNCRIP pulldown. **C–E**. *SIRT1* mRNA stability assay after actinomycin D (ActD) treatment over time, which shows the effects of SYNCRIP depletion on *SIRT1* mRNA degradation. Two-way ANOVA with Šidák's multiple comparisons was used (n = 3, *, $P < 0.05$; **, $P < 0.01$; ***, $P < 0.001$.). All data are presented as the mean ± SD.

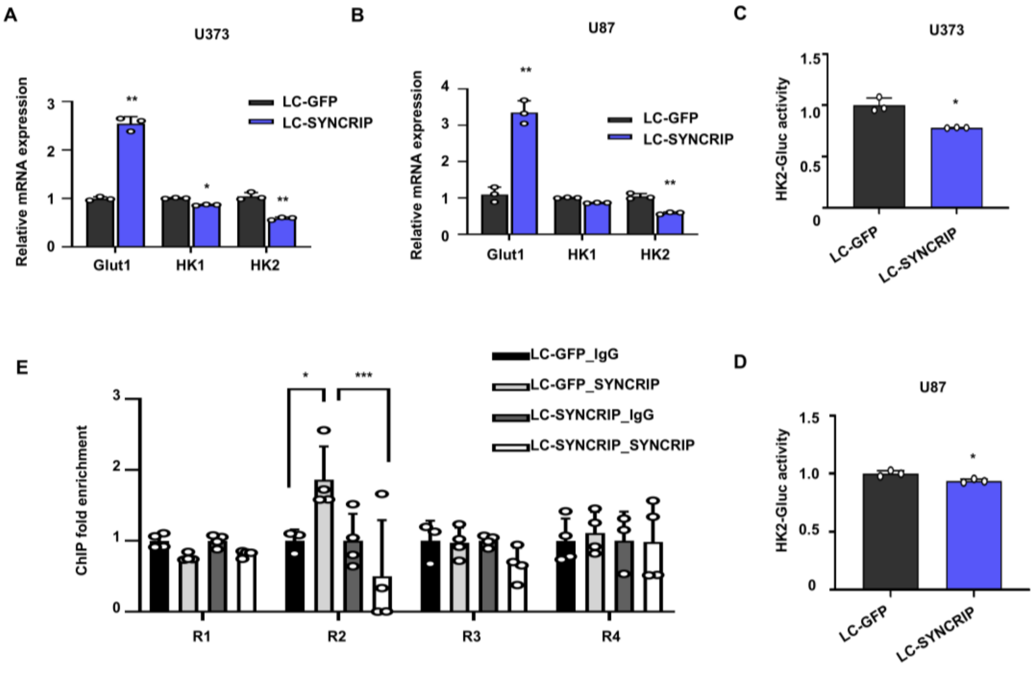
A
U373
Relative mRNA expression
LC-GFP
LC-SYNCRIP
Glut1
HK1
HK2
B
U87
Relative mRNA expression
LC-GFP
LC-SYNCRIP
Glut1
HK1
HK2
C
U373
HK2-Gluc activity
LC-GFP
LC-SYNCRIP
E
ChIP fold enrichment
LC-GFP_IgG
LC-GFP_SYNCRIP
LC-SYNCRIP_IgG
LC-SYNCRIP_SYNCRIP
R1
R2
R3
R4
D
U87
HK2-Gluc activity
LC-GFP
LC-SYNCRIP

Figure S10. SYNCRIP regulates HK2 expression through transcriptional control. A–B. An RT-PCR analysis of *GLUT1*, *HK1*, and *HK2* expression in SYNCRIP-deficient and control cells. Two-way ANOVA with Šidák's multiple comparisons was used (n = 3, *, $P < 0.05$; **, $P < 0.01$). **C–D**. A Gaussia luciferase (Gluc) reporter assay was performed using HK2 promoter constructs in SYNCRIP-deficient and control cells. Gaussia luciferase (Gluc) activity was quantified as the FLUC/RLUC ratio. Student's t-test was used to assess statistical significance (n = 3, *, $P < 0.05$). **E**, Chromatin immunoprecipitation (ChIP) analysis was performed in SYNCRIP-deficient HS683 cells to examine SYNCRIP binding to the HK2 promoter. Two-way ANOVA with Šidák's multiple comparisons was used (n = 4, *, $P < 0.05$; ***, $P < 0.001$.). All data are presented as the mean ± SD.

Table S1. Sequences of primers utilized for quantitative RT-qPCR.

Gene Name		Sequence (5′ to 3′)
hRPL32	Forward	AACCCAGAGGCATTGACAAC
	Reverse	GTTGCACATCAGCAGCACTT
hGLUT1	Forward	AAGTCCTTTGAGATGCTGATCCT
	Reverse	AAGATGGCCACGATGCTCAGATA
hHK1	Forward	GGACTGGACCGTCTGAATGT
	Reverse	ACAGTTCCTTCACCGTCTGG
hHK2	Forward	CAAAGTGACAGTGGGTGTGG
	Reverse	GCCAGGTCCTTCACTGTCTC
hSIRT1	Forward	TAGCCTTGTCAGATAAGGAAGGA
	Reverse	ACAGCTTCACAGTCAACTTTGT
hSOD2	Forward	GCTCCGGTTTTGGGGTATCTG
	Reverse	GCGTTGATGTGAGGTTCCAG
hCAT	Forward	CGGACATGGTCTGGGACTTC
	Reverse	AACTGCCTCCCCATTTGCAT
hPRDX3	Forward	GCCGTTGTCAATGGAGAGTT
	Reverse	TCCACTGAGACTGCGACAAC
hPRDX5	Forward	GCAGCAAGACGGTACAGTGA
	Reverse	GCCAGTCACAAAGGCATCAT
hGCLC	Forward	GGCACAAGGACGTTCTCAAGT
	Reverse	CAGACAGGACCAACCGGAC

hNQO1	Forward	CCTGCCATTCTGAAAGGCTGGT
	Reverse	GTGGTGATGGAAAGCACTGCCT
hZEB1	Forward	AGGATGACCTGCCAACAGAC
	Reverse	TCTGCATCTGACTCGCATTC
hTWIST1	Forward	GCCAGGTACATCGACTTCCT
	Reverse	CCAGCTCCAGAGTCTCTAGA
hSMAD2	Forward	GGGCAGGAAGAAAAGTGGTG
	Reverse	AGGCCTGTTGTATCCCACTG
hMMP2	Forward	GGCATTCAGGAGCTCTATGG
	Reverse	GAGCGATGCCATCAAATACA
hMMP9	Forward	GCTGGCAGAGGAATACCTGTAC
	Reverse	CAGGGACAGTTGCTTCTGGA
